# Prenatal maternal stress: triangulating evidence for intrauterine exposure effects on birth and early childhood outcomes across multiple approaches

**DOI:** 10.1186/s12916-024-03834-w

**Published:** 2025-01-21

**Authors:** Ingunn Olea Lund, Laurie J. Hannigan, Helga Ask, Adrian D. Askelund, Laura Hegemann, Elizabeth C. Corfield, Robyn E. Wootton, Yasmin I. Ahmadzadeh, George Davey Smith, Tom A. McAdams, Eivind Ystrom, Alexandra Havdahl

**Affiliations:** 1 PsychGen Center for Genetic Epidemiology and Mental Health, Norwegian Institute of Public Health, Oslo, Norway; 2 Department of Child Health and Development, Norwegian Institute of Public Health, Oslo, Norway; 3 Department of Psychology, University of Oslo, Oslo, Norway; 4 Nic Waals Institute, Lovisenberg Diaconal Hospital, Oslo, Norway; 5 MRC (Medical Research Council) Integrative Epidemiology Unit, University of Bristol, Bristol, UK; 6 PROMENTA, Department of Psychology, University of Oslo, Oslo, Norway; 7 Population Health Sciences, Bristol Medical School, University of Bristol, Bristol, UK; 8 School of Psychological Science, University of Bristol, Bristol, UK; 9 Social, Genetic and Developmental Psychiatry Centre, Institute of Psychiatry, Psychology and Neuroscience, King’s College London, London, UK

**Keywords:** Maternal stress, Prenatal exposure, MoBa, Emotional problems, Behavioral problems, Internalizing problems, Externalizing problems, Polygenic scores, MBRN

## Abstract

**Background:**

Maternal stress during pregnancy may impact offspring development via changes in the intrauterine environment. However, genetic and environmental factors shared between mothers and children might skew our understanding of this pathway. This study assesses whether prenatal maternal stress has causal links to offspring outcomes: birthweight, gestational age, or emotional and behavioral difficulties, triangulating across methods that account for various measured and unmeasured confounders.

**Methods:**

We used data from the Norwegian Mother, Father, and Child Cohort Study (MoBa), including maternal reports on prenatal stress at work, at home, and via stressful life events as exposures. Outcomes were children’s birthweight and gestational age, from the Medical Birth Registry of Norway, and maternal reports on early offspring emotional and behavioral difficulties. We assessed associations using four approaches: sibling control analyses, gene-environment interaction analyses, intergenerational Mendelian randomization (MR), and negative control (i.e., postnatal stress) analyses.

**Results:**

Maternal prenatal stress was observationally associated with offspring lower birthweight (e.g., β_work_ = − 0.01 [95%CI: − 0.02, − 0.01]), earlier birth (e.g., β_work_ = − 0.04 [95%CI: − 0.04, − 0.03])), and more emotional (e.g., β_events_ = 0.08 [95%CI: 0.07, 0.09]) and behavioral difficulties (e.g., β_relationship_ = 0.08 [95%CI: 0.07, 0.09]) in the full sample (*N* = 112,784). However, sibling control analyses (*N* = 36,511) revealed substantial attenuation of all associations after accounting for familial factors. Gene-environment interaction models (*N* = 76,288) showed no clear evidence of moderation of associations by mothers’ polygenic scores for traits linked to stress sensitivity. Intergenerational MR analyses (*N* = 29,288) showed no clear evidence of causal effects of maternal plasma cortisol on any offspring outcomes. Negative control exposure analyses revealed similar effect sizes whether exposures were measured prenatally or postnatally.

**Conclusions:**

Our results indicate that links between prenatal maternal stress and variation in early offspring outcomes are more likely to be confounded than causal. While no observational study can rule out causality, the consistency of our findings across different approaches is striking. Other sources of prenatal stress or more extreme levels may represent intrauterine causal risk factors for offspring development. Nonetheless, our research contributes to identifying boundary conditions of the fetal programming and developmental origins of health and disease hypotheses, which may not be as universal as sometimes assumed.

**Supplementary Information:**

The online version contains supplementary material available at 10.1186/s12916-024-03834-w.

## Background

More than one in four individuals globally experience a psychiatric disorder at some point in their lifetime [1], making mental health challenges one of the most critical public health concerns of our time. A growing body of research suggests that maternal stress during pregnancy is linked to early-life development and behavior difficulties. This association is often framed within the context of fetal programming [2–4] and the developmental origins of health and disease (DOHaD) [5], which suggest that in utero experiences, such as exposure to maternal stress, can have both short and long-lasting impacts on a child’s early development, including birthweight, gestational age [6–8], and mental health outcomes [9, 10]. However, fetal programming is just one of several potential mechanisms that could explain this association. Intergenerationally shared genetic predispositions and environmental factors complicate our understanding of the relationship between maternal stress during pregnancy and any child outcomes. Rigorous, multidisciplinary research is essential for teasing apart the contributing factors to these relationships. Identifying genuine causal risk factors may pave the way for creating evidence-based interventions aimed at reducing the prevalence and severity of mental health challenges.

Ethical and practical barriers prevent conducting randomized controlled trials with randomization of pregnant women to be exposed to varying levels of stress for research purposes. As such, the field relies on observational data. Several prospective, longitudinal studies have found an association between maternal stress, depression, and anxiety in pregnancy and various emotional and behavioral outcomes in offspring, see, e.g., [11–20]. For example, one US study that analyzed data from three different pregnancy cohorts found a relationship between maternal stress during pregnancy and emotional issues in offspring observed at ages 8 to 9 years [15]. While many studies originate from Europe, North America, and Australia, similar findings have been reported in other regions [13, 21–25]. In one South African study, 3-year-old children born to mothers experiencing both depression and anxiety during pregnancy faced a higher risk of emotional difficulties compared to their counterparts born to mothers experiencing only one of these issues; children born to mothers without these symptoms were found to be at the lowest risk [22]. Overall, across different data sources and populations, the evidence for an observational association between maternal prenatal stress and offspring outcomes is quite consistent [26].

Existing observational research has established a correlation between maternal stressors and emotional and behavioral outcomes in children, but most studies cannot determine whether this association is causal. Natural experiments offer somewhat more convincing evidence for potential causal pathways. For instance, being exposed in utero to natural disasters, such as floods, storms, and earthquakes seems to be linked to postnatal offspring outcomes, potentially suggesting a causal impact of disaster-related maternal stress [27–30]. Such studies support the notion that direct pathways between prenatal stressors and long-term outcomes in offspring exist. However, natural experiments also come with limitations. The multi-faceted nature of the stressful exposures exploited in these studies leave open numerous routes for potential confounding. Many of the studies are relatively small and without clearly defined control groups. To precisely determine the effect of natural disasters on pregnancy and child outcomes, large, diverse samples with well-defined control groups and extended longitudinal research designs are necessary [31]. Moreover, results relating to unique and extreme environmental stressors may not generalize well to forms of emotional stress more commonly experienced by women during pregnancy.

Establishing whether in utero exposure to more typical prenatal emotional stress has a causal effect on child development requires ruling out potential confounding pathways. One such pathway is the frequent correlation between in utero and postnatal exposure; that is, children exposed to maternal stress and mental health issues prenatally are also more likely to be exposed during the early years of their life, making it challenging to distinguish the unique effects of in utero stress from postnatal influences [32]. Stress and mental health issues in mothers of young children could potentially affect their children’s emotional and behavioral development through various mechanisms, such as reduced emotional availability from a stressed or depressed mother, which in turn could impact a child’s sense of emotional security and attachment. For evidence to support a causal effect of prenatal maternal stress, the potential effects of these postnatal mechanisms must be accounted for. Some studies have included information on maternal stress and mental health both during pregnancy and postnatally [16, 33–36]. For example, a US study found that even after accounting for maternal mental health when the children were 4 years old, exposure to stressful life events and heightened perceived stress during pregnancy remained independent predictors of emotional and behavioral difficulties in offspring [16]. Another US study showed that prenatal stress and depressive symptoms were associated with emotional and behavioral issues in children aged 4 to 6 years, even when controlling for maternal stress and depressive symptoms when the child was assessed [17]. A recent systematic review and meta-analysis showed that while prenatal and postnatal distress are moderately correlated and both significantly associated with offspring behavior difficulties, the effect of prenatal distress on offspring behaviors was essentially unchanged after adjusting for postnatal distress (from *r* = 0.160 to *r* = 0.159) [26]. Overall, these findings underscore the links between prenatal distress and behavior difficulties while also highlighting the need to consider both prenatal and postnatal periods in research and intervention strategies.

Another important pathway by which observational associations between prenatal maternal emotional stress and offspring outcomes may be confounded is via direct genetic transmission. Mothers share 50% of their genes with their biological children. To the extent that the same genetic variants linked to maternal traits—for example, the exposure to [37] or influence of [38] stressful events—also directly influence child outcomes, this opens a route by which observational links between these traits can be spuriously inflated. Since recent evidence has accumulated to show that common genetic variants have largely non-specific and highly overlapping associations with complex behavioral traits [39–41], this pathway is highly likely to be relevant for links between prenatal stress and offspring emotional and behavioral outcomes. Aside from a few notable exceptions [42–47], most studies have not employed causally informative and genetically informed designs to explore the associations between prenatal stress, including mental health factors, and offspring emotional and behavioral outcomes. Most studies controlling for this pathway suggest either a lack of association between prenatal exposure and child outcomes [45, 48], or that association strength depends on the child’s genetic susceptibility [42–44]. Several of these studies suggest that prenatal exposure to stress, anxiety, or depression does not directly cause emotional and behavioral difficulties in offspring [43–45, 48]. For instance, studies showing that maternal polygenic risk scores for emotional, behavioral, and neurodevelopmental conditions are associated with prenatal exposure to stressful life events and prenatal anxiety and depression, suggesting that associations between such prenatal exposures and child emotional, behavioral, and neurodevelopmental outcomes are at least partly genetically confounded [43, 49].

Further, three large Norwegian studies, using data from the Norwegian Mother, Father, and Child Cohort Study (MoBa), used sibling comparison and negative control analyses to explore these associations [44, 45, 50]. One of these studies examined the association between maternal and paternal prenatal anxiety and offspring behavior difficulties at ages 1.5 and 5 years. While observational findings suggested an association between prenatal exposure to maternal anxiety and offspring behavior difficulties, results were reduced after controlling for unmeasured family factors in sibling comparisons. This indicated that the associations were primarily attributable to genetic or shared environmental factors rather than direct effects of prenatal stress [44]. Another pair of MoBa studies, which looked at both prenatal and postnatal depression, found that only concurrent maternal depression had a significant impact on offspring outcomes after sibling comparison [45], and confirmed the importance of a genetic transmission pathway in the observational association in a children-of-twins design [47]. These latter studies highlight the utility of large family-based cohorts like MoBa, which allow for stringent control for genetic confounding.

Different causally informative and genetically informed designs have different strengths, but also different vulnerabilities to biases. For instance, sibling control analyses control for siblings’ shared genetic and environmental factors, thus isolating the effect of specific variables. This allows for comparing outcomes within siblings who are similar or different on the exposures of interest [51]. However, bias can occur from environmental factors and experiences not shared between siblings [52]. Gene-environment interaction analyses can uncover how genetic predispositions and environmental factors interact to affect outcomes [53]. However, if gene-environment correlations are not properly considered, bias may occur. Intergenerational Mendelian randomization (MR) [54] uses genetic variants as instrumental variables to infer causal relationships, reducing confounding biases. However, this approach assumes that the genetic instrument only affects the outcome through exposure, which may not be true. Negative control analyses [55, 56] identify and control for unmeasured confounding by replacing either exposures or outcomes with equivalent variables for which the causal mechanism under study should not apply. However, if negative control variables are not appropriately chosen, this can lead to incorrect causality assumptions. A prospective triangulation approach [57] that employs various designs, ideally with different underlying assumptions, limitations, and biases, can enhance the robustness of findings. If diverse methods produce results pointing in the same direction, it significantly bolsters the credibility of the conclusions. Ultimately, triangulation [58]—whether in a single study or in the literature over time—is the most typical way research questions are definitively answered.

Here, we attempt to robustly answer whether maternal stress during pregnancy represents a causal risk factor for offspring’s emotional and behavioral difficulties by adopting a triangulation approach. We include birthweight and gestational age as a form of positive control [6–8, 59]. These outcomes are close to the exposure and are close to error-free measures. Additionally, they are influenced by established prenatal causal risk factors, such as smoking [60]. This makes them ideal for detecting the potential effects of maternal stress. Using data from the MoBa cohort and linked birth registry information, we use four distinct analytical approaches to address the research question. Specifically, we carry out (1) sibling control analyses, which estimate exposure-outcome associations with and without the effects of familial confounding; (2) genotype-environment (GxE) interaction analyses, which test indirectly for causal links by examining moderation of the intergenerational pathways; (3) intergenerational Mendelian randomization analyses, which estimate the association between maternal stress—instrumented by maternal genetic variants linked to plasma cortisol—and child outcomes; and (4) negative control analyses, which re-estimate target associations using “exposures” that occur after outcomes. We interpret results from these approaches to evaluate the plausibility of a causal mechanism underlying associations between maternal stress exposure during pregnancy and early developmental outcomes in offspring.

## Methods

### Study sample

The Norwegian Mother, Father, and Child Cohort Study (MoBa) is a population-based pregnancy cohort study conducted by the Norwegian Institute of Public Health [61]. Participants were recruited from all over Norway from 1999 to 2008. Invited women consented to participation in 41% of the pregnancies. The cohort includes approximately 114,500 children, 95,200 mothers, and 75,200 fathers. The current study is based on version 12 of the quality-assured data files released for research in January 2019 and updated to reflect participant withdrawals as of January 2024. The establishing of MoBa and initial data collection was based on a license from the Norwegian Data Protection Agency and approval from The Regional Committees for Medical and Health Research Ethics. The MoBa cohort is currently regulated by the Norwegian Health Registry Act. The current study was approved by The Regional Committees for Medical and Health Research Ethics (2016/1702).

We also used data from the Medical Birth Registry of Norway (MBRN), a national health registry containing information about all births in Norway.

Blood samples were obtained from both parents during pregnancy and from mothers and children (umbilical cord) at birth [62, 63]. Genetic information (*N* = 6,981,748 single nucleotide polymorphisms [SNPs]) was available for 76,465 MoBa children, 77,387 mothers, and 50,462 fathers, all of European ancestry, after post-imputation quality control [64].

### Measures

For a detailed overview of the items used in the assessments of maternal stress and child behavioral and emotional problems, see sTable 1 in Additional file 1.

#### Maternal stress

Mothers’ prenatal stress was assessed—for each of the pregnancies with which they participated in MoBa—via self-report, with selected items covering potential stressors from different aspects of expectant mothers’ environments. These included ratings of stress at work, (dis)satisfaction and stress in their partner relationship, and the number of stressful life events experienced in the past year.

Stress at work: At 15 weeks’ gestation, mothers rated eight statements covering the extent to which they considered their current work stressful and how often they felt overwhelmed with work tasks on a four-point scale. Four were specifically related to stress (e.g., “My work is very stressful”) and were selected for these analyses. Previous studies based on MoBa have used the scale [11], or items from the work stress scale [65].

Relationship stress: Mothers completed a 10-item scale assessing relationship satisfaction with six-point response format at 15 weeks’ and 30 weeks’ gestation [66]. This scale measured the frequency of disagreements with partners, their overall happiness with the relationship, and perceptions about their partner’s happiness in the relationship [67]. Scores on the two relationship satisfaction outcomes were averaged.

Adverse life events: At 30 weeks’ gestation, mothers responded to several items about their exposure to recent adverse life events (< 12 months), such as the death of a close friend or relative, involvement in accidents, and financial problems. This was quantified using a 0–9 count variable. This scale was developed for MoBa, inspired by Coddington [68], and adapted to adult respondents.

#### Offspring outcomes

##### Birth registry outcomes

We obtained data on offspring birthweight and gestational age via linkage to the Norwegian birth registry. These measures were selected as the offspring outcomes measured nearest in time to the intrauterine exposure and therefore likely among the most sensitive indices of any intrauterine effects. Literature suggests that both birthweight and gestational age may be susceptible to changes in the intrauterine environment [6–8] and predictive of behavioral and emotional difficulties later in development [69–72].

##### MoBa questionnaire outcomes

To evaluate symptoms of emotional and behavioral difficulties in offspring, we used maternal reports of items from the internalizing and externalizing domains of the Child Behavior Checklist (CBCL) [73] when children were aged 1.5, 3, and 5 years. Mothers rated a series of statements about their child’s behavior on a scale with the following response options: “Not true,” “Somewhat or sometimes true,” or “Very true or often true.” Due to limited space in the MoBa questionnaires, item selection was necessary. A consensus among clinical and developmental psychology experts guided this process. Both the offspring emotional difficulties measure (5 items at 1.5-year wave, 9 items at 3-year wave, 11-items at 5-year wave; ordinal Cronbach’s alpha range 0.66–0.85) and the behavioral difficulties measure (8/11/11 items; 0.69–0.83) showed acceptable internal consistency. The CBCL for older children is validated in a Norwegian general population sample [74] and versions for younger children in Dutch and Danish contexts [75, 76]. The CBCL version in MoBa has been used in a range of studies, e.g., [50, 77–79].

We calculated scale variables by averaging scores of answered items and multiplying by the total number of items in the scale. Scores for individuals with > 50% item-level missingness for a given scale were set to missing.

#### Covariates

Information about children’s biological sex at birth and parity (the number of children previously born to each mother) was obtained from the MBRN.

#### Polygenic scores

Polygenic scores (PGS) are calculated using effect estimates for all variants in common between a discovery sample (i.e., genome-wide association studies; GWAS) and target sample, for variants whose effects in the GWAS had a *p* value below a specified threshold. We calculated polygenic scores based on summary statistics from GWAS of three traits that may moderate mothers’ responses to stress during pregnancy. These were neuroticism [80], post-traumatic stress disorder (PTSD) [81], and ADHD [82]. As in a previous study [79], these traits were chosen due to their association with heightened sensitivity to environmental stressors—ADHD is linked to an increased feeling of being overwhelmed by environmental stimuli, neuroticism to excessive worrying, and PTSD to severe reactions to traumatic events. We also included height [83] as a negative control, assuming that height-associated variants are unlikely to directly influence maternal stress response mechanisms. We created all PGS using PRSice2 [84] using a clumping and thresholding approach (250 kb window, *p* = 1, *r*^2^ = 0.1). The *p* value thresholds for SNP inclusion were 5 × 10^−8^, 10^−7^, 10^−6^, 10^−5^, 10^−4^, 0.001, 0.01, 0.05, 0.1, 0.5, and 1. We adjusted each score for the effects of variables produced during the quality control process (genotyping batch) and the first 20 principal components to account for structural confounding. To optimize the predictive power of our polygenic scores while avoiding overfitting, we used a polygenic score-principal component analysis (PGS-PCA) approach, as outlined in [85]. This approach involves extracting the first principal component of scores across all the *p* value thresholds (within a trait) for analysis. PGS with a corresponding trait measured in MoBa mothers (neuroticism, ADHD) were validated in the sample.

### Analyses

**Fig. 1:**
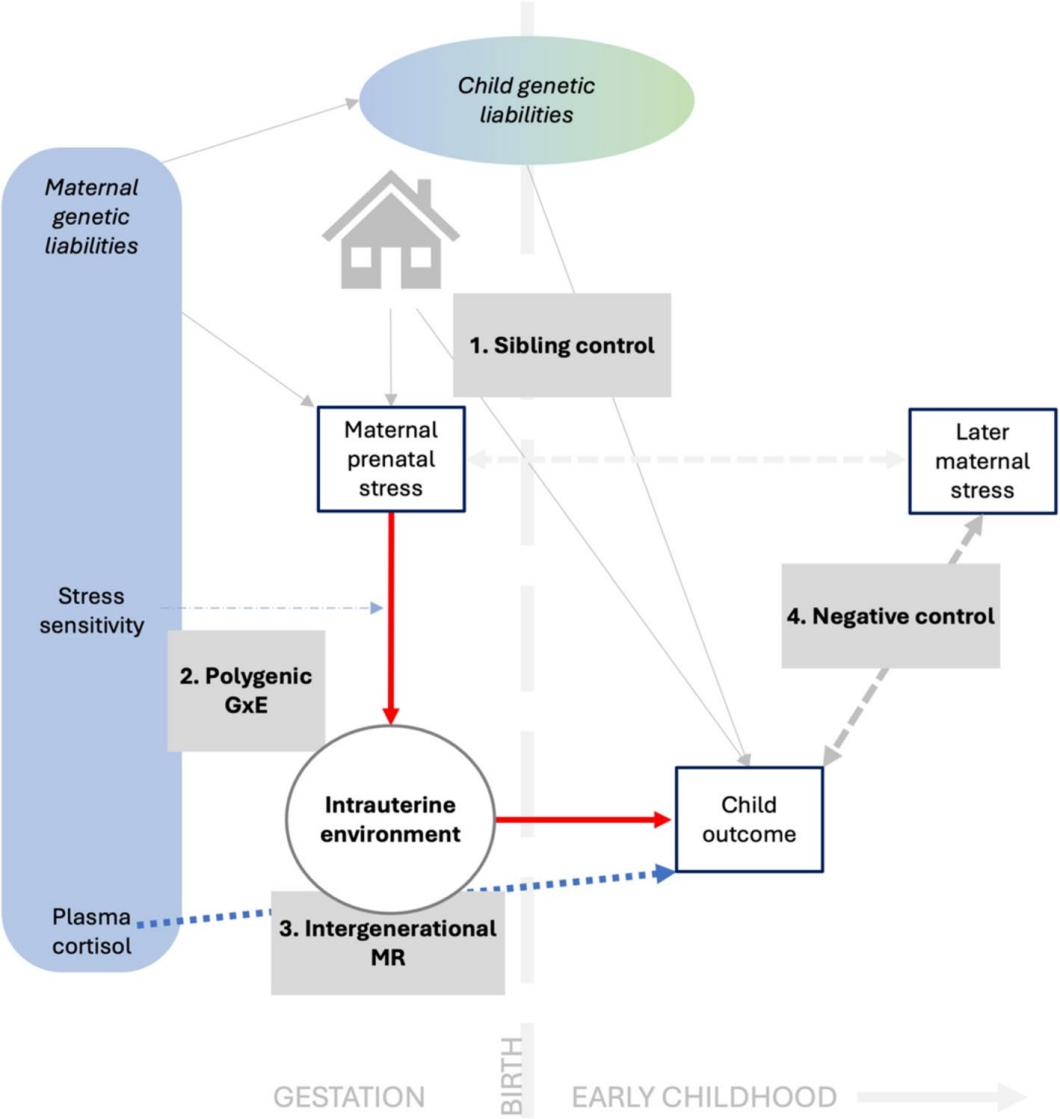
Study schematic: investigating maternal prenatal stress effects on offspring using four methods Note: thick red arrows indicate the central hypothesis under investigation (the intrauterine environment is inferred rather than directly measured); gray boxes place each analytic approach proximate to the pathways they estimate or adjust for in order to test the central hypothesis: sibling control models (1) adjust for the paths shown by thin solid arrows; polygenic GxE analyses (2) estimate the moderation pathway indicated by the thin dashed arrow; intergenerational MR analyses (3) estimate the pathway indicated by the thick dotted arrow; negative control analyses (4) estimate the (non-causal) pathway indicated by the thick, dashed, double-headed arrow

An overview of the analytical strategy used in the study is shown in Fig. 1. We ran analyses in four distinct components, each designed to contribute evidence that could be triangulated with respect to whether maternal prenatal stress causally influences offspring development. These components are described, in turn, in the text below.

#### Sibling control analyses

We used multilevel structural equation modeling (SEM) to estimate associations between maternal prenatal stress exposure and child outcomes with adjustment for familial confounding. The basic premise of the multilevel SEM approach is to allow a theoretical model of the relationships between different observed and latent variables to be parameterized in terms of their variance/covariance structure in data where individual observations are clustered (in our case, in nuclear families). Specifically, our multilevel SEM approach (see sFigure 1 in Additional file 1 for an illustrative path diagram) differentiates “between” level effects from “within” level effects. Between-level effects are the portion of the association between prenatal stress and child outcome shared between siblings and thus consistent irrespective of the variations of stress reported during each pregnancy. Within-level effects capture the extent to which sibling differences in exposure to prenatal stress predict sibling differences in child outcomes. If the association between prenatal stress and an outcome is causal, within-family effects should be present.

We restricted these analyses to a sub‐sample of MoBa families with at least two participating siblings (*N* = 36,511 individuals born to 17,569 mothers) and data available on at least one outcome or exposure. We first ran a model for each outcome, including all three exposures, estimating the observational exposure-outcomes association in the sibling sub-sample by constraining all between-level effects to zero. For outcomes with measures at multiple waves (i.e., offspring emotional and behavioral difficulties), we included all three measurement occasions in the same model, allowing residual variances to correlate across time. Next, we ran adjusted versions of the same models, this time allowing exposure-outcome associations to be appropriately partitioned according to the extent to which they were attributable to familial confounding (i.e., consistent for a mother, across pregnancies) versus consistent with causal links (i.e., specific to each member of a sibling pair). We compared the estimates from the two models, with the interpretation that attenuation of estimated effects between the first and second models was indicative of the extent to which observational associations were subject to confounding.

Parity and biological sex of the child were included as covariates at the within-level of all models. Additionally, to ensure our sibling sub‐sample was representative of the overall sample, we used inverse probability weighting based on the characteristics from the larger MoBa sample, as described in [86]. Further details of this are included in Additional file 1 (sMethods1 and sFigures 2–4).

#### Polygenic GxE analyses

We conducted polygenic genotype-environment interaction (GxE) analyses using the entire analytic sample of MoBa children (both singletons and siblings, with any relevant data available: *N* = 93,564 individuals). We ran multiple linear regression models with every exposure for each outcome, including measures from all three waves for the offspring’s emotional and behavioral outcomes, respectively, in a single structural equation model. See sMethods 2 and sEquation 1 in Additional file 1 for the logic and formal specification of the basic model. For each model, we ran a version with terms for the main and moderating effects of one of the four PGS. Overall, this meant that we ran 16 models (four outcomes × four moderators), with a total of 96 relevant exposure-PGS interaction effects (one per each of the three exposures in the eight models with birthweight and gestational age as outcomes and three for each of the three exposures in the eight models with offspring emotional and behavioral difficulties as outcomes, due to these being measured on three occasions).

To reduce the multiple testing burden, we tested whether the exposure-PGS interaction effects for offspring emotional and behavioral difficulties could be constrained to be equal across the various measurement waves. If they could, a single effect was estimated (potentially reducing the number of effects to 48, if all constraints were accepted). Then, a Benjamini-Hochberg [87] false discovery rate (FDR) correction was applied across the final list of all interaction effects for interpretation. Interaction effects that were significant after multiple testing corrections would be interpreted as evidence consistent with a causal relationship between the exposure and outcome in question (see sMethods 2 in Additional file 1 for an expansion of this logic).

Covariates such as parity and biological sex of the child, including their interaction terms with the exposures and moderators, were included in all models. To handle missing data, we used the full information maximum likelihood estimation. Additionally, we estimated cluster-robust standard errors to account for data dependencies due to the inclusion of siblings.

#### Intergenerational Mendelian randomization (MR) analyses

To seek evidence of intrauterine effects of maternal prenatal stress that are orthogonal to the exposure-outcome associations used in the previous components, we conducted intergenerational MR analyses [54], with maternal stress instrumented by SNPs linked to plasma cortisol (sFigure 5 in Additional file 1). The GWAS of plasma cortisol [88], conducted in non-pregnant individuals of both sexes, reported four SNPs with significant effects on plasma cortisol at the genome-wide level. We had information available on three of these SNPs (*rs9989237*, *rs2736898*, *rs7146221*) in MoBa and found a proxy (*rs7141205*) in high linkage disequilibrium (*R*^2^ > 0.95) for the fourth (*rs11620763*). In a subset of the analytic sample containing only unrelated trios with genotype data available (*N* = 29,288 individuals), we estimated the effects of the maternal variant of each SNP on each of our outcomes while controlling for effects from both the child’s and father’s genotype [88], the child’s sex at birth, and technical covariates (20 principal components and genotyping batch). As a sensitivity analysis, since control for the child’s genotype could “over-adjust” and render null a small effect of maternal SNPs, we also ran a version of these models controlling for the technical covariates only. Using the TwoSampleMR [89] and MendelianRandomization packages [90] in R, the SNP effects were combined per outcome in an inverse variance-weighted meta-analytic estimate, with non-independence among SNPs accounted for by incorporating a linkage disequilibrium matrix from the 1000 genomes EUR reference panel (as described in TwoSampleMR [89] documentation). From the intergenerational MR analyses, non-zero effects of maternal plasma cortisol-linked genetic variants on child outcomes would be consistent with causal intrauterine effects of maternal prenatal stress. See sFigure 6 in Additional file 1 for a directed acyclic graph (DAG) that illustrates the MR design.

#### Negative control analyses

Negative control analyses work by facilitating the comparison of estimates of a given observational effect with estimates of a similar effect obtained in a scenario where the causal mechanism of interest is implausible [55, 56]. This can be done, for example, by switching out an exposure of interest with a variable that shares possible confounders of the relationship but does not represent a valid exposure for the outcome in question. In this final component of our analyses, we leveraged the longitudinal structure of the MoBa sample, including the entire analytic sample (*N* = 112,784—see results for data availability for specific exposures and outcomes). Using two series of linear regression models, we compared estimates of the association between prenatal maternal stress and our outcomes with those obtained when the exposure (maternal stress) was experienced after the outcome in question (i.e., when birthweight was the outcome, negative control models could include stress measured at 1.5, 3, and 5 years, whereas for offspring emotional difficulties measured at 3 years, only maternal stress measured at 5 years was considered a valid negative control). These analyses included only relationship stress and life events as exposures, since work-related stress was not measured postnatally in MoBa. When comparing the effect sizes from the two sets of models, stronger associations in the models with prenatal exposures vs. those with negative control exposures would be expected if there is a causal intrauterine effect of prenatal maternal stress.

#### Software and analytic code

Most of the analyses were performed in R (version 4.1.2) [91], with multilevel structural equation models carried out using Mplus version 7.31 [92] via R using the *mplusAutomation* [93] package. The following R packages were also used in the project: lavaan [94] 0.6.17; mice [95] 3.16.0; phenotools [96] 0.3.2; psych [97] 2.4.3; tidyverse [98] 2.0.0; weights [99] 1.0.4. All R code for the project and input files for Mplus are available at https://github.com/psychgen/maternal-prenatal-stress.

## Results

### Descriptive statistics, demographic characteristics, and selection effects

Descriptive statistics for the main study variables are presented in Table 1. Demographic information about the sample, including biological sex of the child, parity, and maternal years of education and income, are presented in sTable 2 in Additional file 1. The sample exhibited some selective attrition, with outcome availability correlated with prenatal stress exposure. However, in all cases these effects were minimal, with prenatal stress scores rarely differing by more than one tenth of a standard deviation between those with and without outcome data available (see sTable 3).

**Table 1:** Descriptive statistics for the exposures, outcomes, and negative control variables used in the analyses

Measure	*N*^a^	Mean	SD	Min	Max
*Exposures*					
Prenatal work stress	93,838	13.952	3.460	6	24
Prenatal stressful life events	94,393	0.946	1.084	0	8
Prenatal relationship stress	97,823	6.800	6.250	0	50
*Offspring outcomes*					
Birthweight (grams)	112,717	3561.657	598.407	–	–
Gestational age (days)	112,323	278.495	13.886	–	–
Emotional problems (1.5 years)	75,626	1.280	1.223	0	10
Emotional problems (3 years)	58,128	2.211	1.976	0	18
Emotional problems (5 years)	41,177	1.970	2.105	0	22
Behavioral problems (1.5 years)	75,821	3.813	2.228	0	16
Behavioral problems (3 years)	58,129	5.508	3.180	0	22
Behavioral problems (5 years)	41,179	3.739	3.056	0	21
*Negative control exposures*					
Maternal stressful life events (1.5 years)	73,797	1.043	1.156	0	11
Maternal stressful life events (3 years)	56,508	0.857	1.106	0	10
Maternal stressful life events (5 years)	40,895	0.786	1.051	0	9
Maternal relationship stress (1.5 years)	72,143	8.235	7.804	0	50
Maternal relationship stress (3 years)	54,300	4.949	4.495	0	25
Relationship stress (5 years)	38,662	4.586	4.516	0	25

^a^*N* here represents the number of unique, non-missing observations for each variable—for maternal variables, these include multiple measures of the same mother where they have participated in MoBa for more than one pregnancy

### Sibling control analyses

**Fig. 2:**
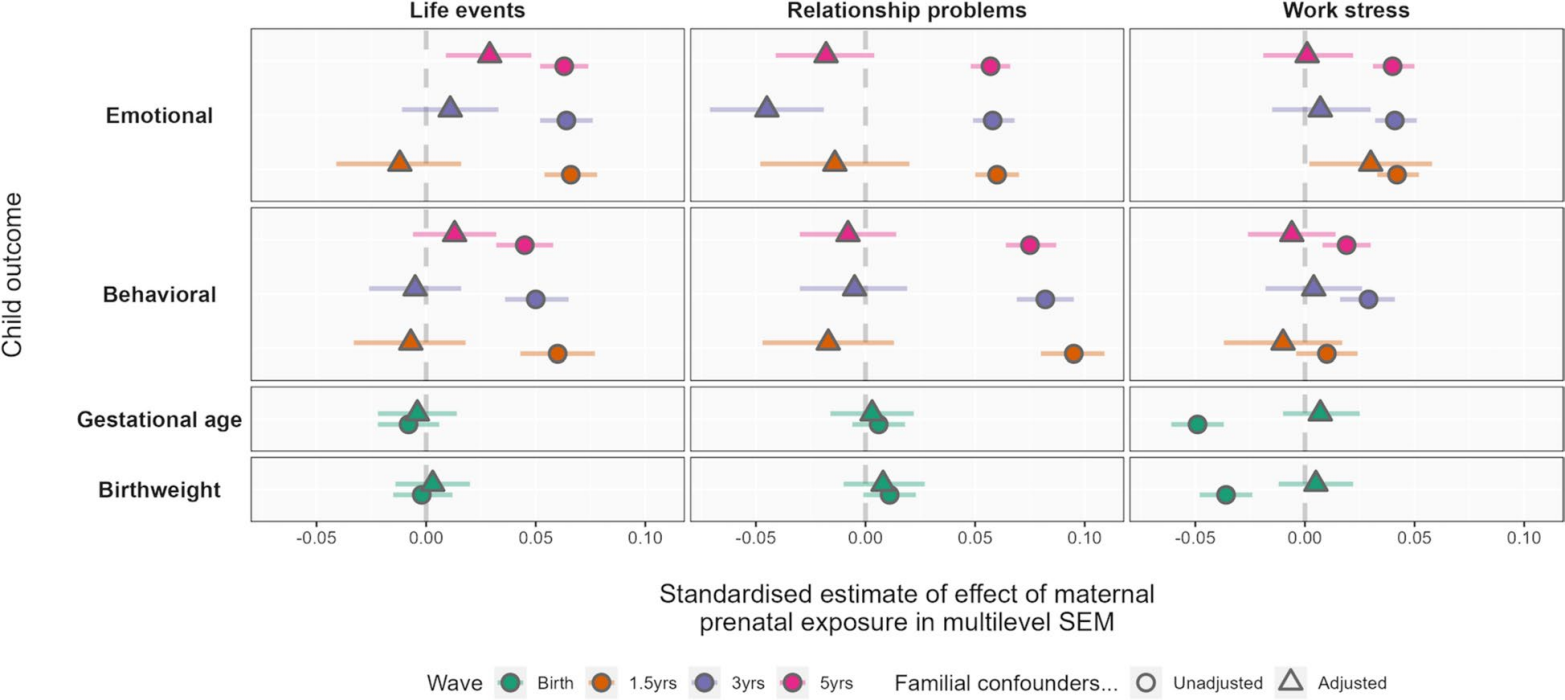
Maternal prenatal stress effects on offspring outcomes before and after sibling comparison adjustment

We conducted sibling control analyses to estimate exposure-outcome associations with and without adjustment for familial confounding. The results of the sibling control analyses are summarized in Fig. 2. In the sibling subset of the MoBa participants, we observed associations between all domains of prenatal stress and all offspring emotional and behavioral outcomes and between work stress and gestational age and birthweight before adjusting for potential familial confounding. Equivalent estimates from the full sample and a comparison of unadjusted observational estimates from the full sample and sibling sub-sample are presented in sFigure 7 in Additional file 1. See sTable 4 for parameter estimates from the observational models in the full sample. After adjusting for shared familial confounding, all effect estimates were substantially attenuated toward the null (triangular markers in Fig. 2), which is inconsistent with causal intrauterine effects.

### GxE analyses

We conducted linear regression analyses with moderation of exposure-outcome associations by maternal polygenic scores linked to environmental sensitivity. We show parameter estimates for the interaction effects from the GxE analyses in sTable 5 in Additional file 1. We observed no moderation by the polygenic scores for any of the associations. This finding is inconsistent with causal intrauterine effects, assuming that the strength of such effects would vary with maternal environmental sensitivity indexed by the polygenic scores.

### Intergenerational Mendelian randomization analyses

We included intergenerational MR analyses to estimate the association between maternal stress—instrumented by maternal genetic variants linked to plasma cortisol—and child outcomes.

**Fig. 3:**
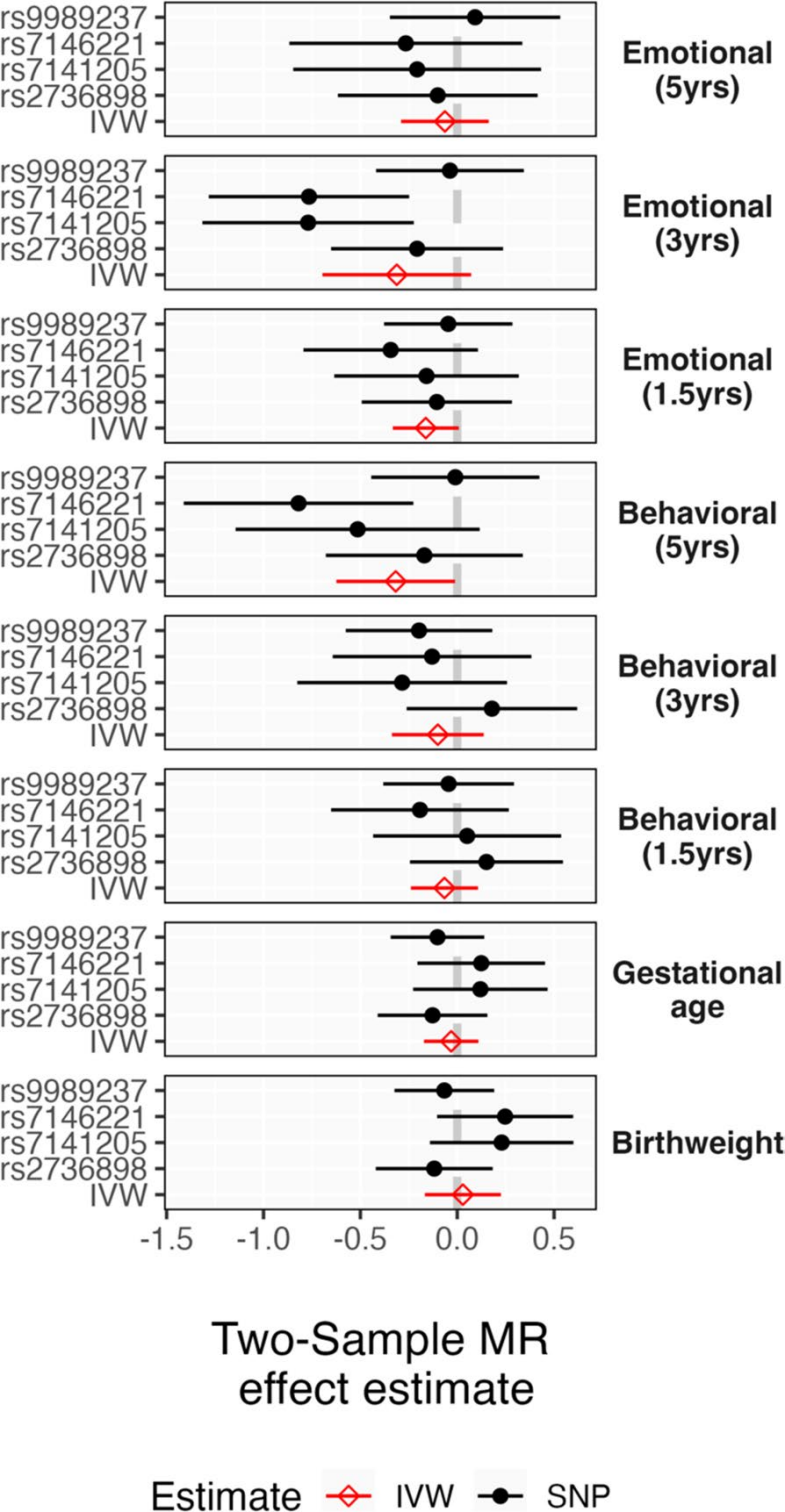
Intergenerational MR estimates of maternal plasma cortisol linked SNPs on offspring outcomes Note: IVW, inverse variance weighted meta-analytic estimate; SNP, single nucleotide polymorphism

Figure 3 shows the results from the MR analyses. None of the maternal plasma cortisol-linked genetic variants, individually or collectively, showed any consistent pattern of association with any of the offspring outcomes. This is inconsistent with causal intrauterine effects of maternal prenatal stress, assuming that maternal cortisol represents a mechanism by which stress influences the intrauterine environment. In sensitivity analyses where SNP-outcome effects were estimated without adjustment for children’s genotypes to account for possible overadjustment bias, the pattern of results (see sFigure 5 in Additional file 1) was very similar.

### Negative control analyses

**Fig. 4:**
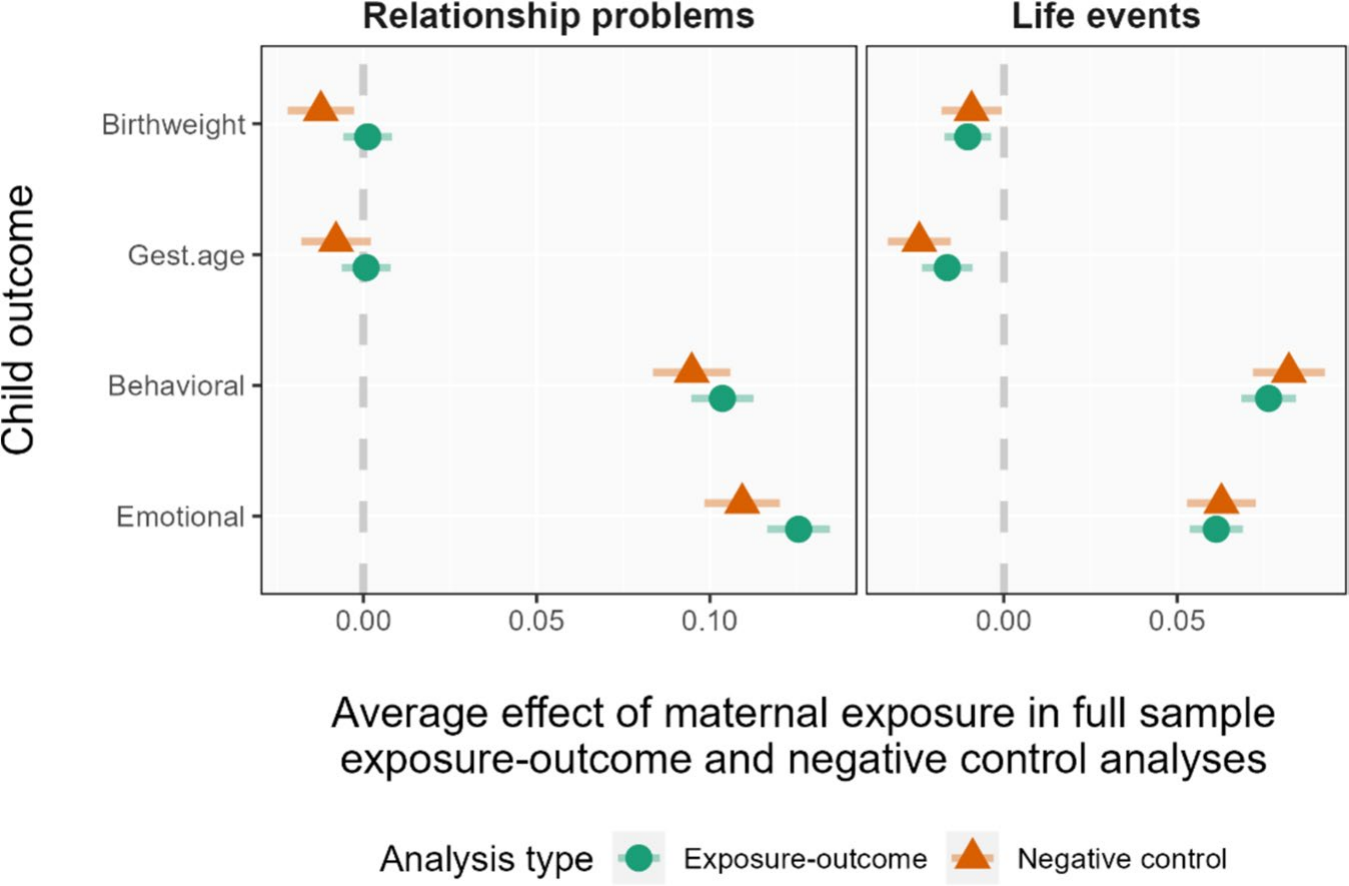
Negative control analysis of the association between maternal stressors and offspring outcomes Note: the figure shows the comparison of estimates from valid exposure-outcome and negative control analyses of the association between two domains of maternal stress (relationship stress and stressful life events) and offspring outcomes

We used negative control analyses to compare estimates from valid exposure-outcome models with those using similar variables, but where the target causal mechanism could not have been involved due to the timing of exposure. Figure 4 shows the results from the negative control analyses. The magnitude of the association in a negative control analysis indicates the extent of potential confounding for the exposure-outcome estimates. Associations between the “exposures” (relationship stress and stressful life events) all outcomes were very similar regardless of whether or not the exposure occurred (or, at least, was measured) prior to the outcome. sTable 6 in Additional file 1 shows parameter estimates from all models in the negative control analyses.

## Discussion

Using a multi-method triangulation approach, we examined the causal links between maternal stress during pregnancy and offspring birthweight, gestational age, and emotional and behavioral difficulties in a Norwegian population cohort. Despite observational associations between maternal stress and offspring outcomes, our comprehensive approach found little evidence consistent with a causal intrauterine exposure effect after accounting for genetic and environmental confounders. The findings suggest that the observed associations may be more attributable to these confounding factors than a direct causal pathway.

Our study’s observational results suggested an association between prenatal stress exposure and various offspring outcomes: gestational age, birthweight, and emotional and behavioral difficulties. This aligns with the findings of many previous studies [11–20, 100]. However, results from our analyses challenge any interpretation of these observational findings as evidence of causal effects [11–20]. Instead, our results are consistent with other studies using genetically informed approaches, which suggest that maternal stressors during pregnancy may not be causally related to developmental outcomes in offspring during preschool years [43–45, 48]. Indeed, our results strengthen the evidence that confounding is the most likely explanation for these links in several ways. First, while most prior studies apply a single method, our comprehensive triangulation approach enhances the robustness of the findings. Second, instead of relying on maternal symptoms of anxiety and depression or their objective exposure to stressful events as proxies for stress as most previous studies have done, we include direct—self-reported—measures of maternal prenatal stress in different contexts (at work, in the relationship, and in life events). Third, to account for the possibility that our measures of offspring emotional and behavioral difficulties were either insufficiently proximal to the exposure—or measured too imprecisely to allow causal effects to be detected—we included birthweight and gestational age as forms of positive control. That is, both outcomes are as close as possible to the exposure in time and are also virtually error-free, meaning that they should provide the best possible opportunity to detect even the most transient causal effects of maternal prenatal stress. Consistent with earlier findings [7, 59], we show that stress during pregnancy is observationally associated with lower birthweight and earlier birth. However, the results of our causally informative analyses indicate that these effects—like those for offspring emotional and behavioral difficulties outcomes—are largely accounted for by confounding.

The fetal programming hypothesis [2–4] and DOHaD are often implicitly accepted in the literature on prenatal exposures and are used to explain the effects of a range of prenatal exposures on a range of offspring’s physical and psychological outcomes [14, 101–103]. The mechanism has strong support for certain outcomes, such as fetal alcohol syndrome [101, 104] where prenatal alcohol exposure is a clear cause. However, a lot of the research that implicitly assumes fetal programming to be true fails to mention genetic confounding. For instance, a recent systematic review and meta-analysis on prenatal stress and offspring behavior difficulties suggested a small but consistent effect that remains after accounting for maternal postnatal distress but does not take genetic confounding into account [26]. Our research contributes to identifying boundary conditions of the fetal programming hypothesis and DOHaD, indicating that they may not be as universal as is sometimes assumed. Specifically, our findings do not support the idea that, in our Norwegian cohort sample, prenatal work, life, or relationship stress causally affects either physical outcomes like gestational age and birthweight or psychological outcomes—emotional and behavioral difficulties—in young offspring. This conclusion is drawn with a high degree of confidence for the exposures and outcomes we have examined, given the triangulation approach we used and the highly consistent nature of the evidence derived from it. We focused mainly on normative stressors and have not examined the impact of extreme stressors, such as those experienced during natural disasters, or the potential effect of severe stressors earlier in life, which have also been linked to a higher risk of adverse outcomes [14, 27–30, 105]. As such, there is still a possibility that the mechanism is real but that the context, level, and type of stress required to engage it may be specific.

Nonetheless, our work serves as a basis for future research to further explore the boundary conditions of the fetal programming hypothesis and DOHaD, challenging researchers and educators to re-examine the prevailing assumptions in this area, consider the specificity and limits of prenatal exposure effects, and acknowledge the presence of potential confounding pathways where they exist. Implementing interventions on uncertain foundations is ineffective and a poor use of public resources. Our study contributes a more nuanced understanding of the factors contributing to children’s emotional and behavioral difficulties. This knowledge is relevant to education, social welfare for people with lived experiences, and healthcare sectors. While it is too early to recommend specific interventions based on our findings, the results should guide future research toward evidence-based interventions. If further evidence supports our findings, there may be a need to revise public health messages that currently emphasize the risks posed by maternal stress during pregnancy on offspring’s emotional and behavioral outcomes. If so, this should be done without deprioritizing maternal mental health during pregnancy. Additionally, healthcare professionals could use this information to reassure mothers concerned about the impact of prenatal stress on their children’s emotional and behavioral health.

### Methodological strengths and limitations

A key strength of this study lies in using a large prospective pregnancy cohort, which combines survey, genotype, and health registry data. Another major advantage is that we used prospective triangulation by design, strategically including multiple analytical methods [58], rather than only relying on the more commonly used retrospective triangulation, where results are compared with existing literature. This approach enhances the robustness of the insights into the associations between maternal stress during pregnancy and offspring’s emotional and behavioral difficulties. We use a sibling control design, which powerfully adjusts for familial confounding but relies upon a highly selected sub-sample of MoBa and can also produce biased estimates when confounders are not shared between siblings [106]. The GxE analyses do not share these limitations. However, in turn, they rely on the assumptions that a genetic sensitivity to the environment would moderate any true causal effect and that this sensitivity is captured by the polygenic scores included—despite these being derived based on the main effects of genetic variants on the traits in question. While our GxE analyses can only provide indirect evidence for a causal effect, our use of intergenerational Mendelian randomization represents a more direct test of the mechanism in question and the well-established strengths of instrumental variable analysis [107]. However, our MR analyses are limited because of scant evidence on how well the cortisol-linked SNPs used predict cortisol in pregnancy. To the extent that this prediction is weaker than in the original cortisol GWAS—and available evidence suggests that it might be reduced by up to 50% [8]—our causal effect estimates using these instruments may be biased toward the null. In addition, the standard assumptions that underlie MR methods (e.g., that SNPs are not associated with the outcome by any pathway other than through the exposure) apply here. These limitations, while not inconsequential, are mitigated by using other designs in parallel. Readers unfamiliar with MR should note that the use of single, robustly associated variants as instrumental variables in our approach is not related to the now outdated theoretically driven selection of candidate genes, which resulted in unreliable and inconsistent findings (particularly for GxE effects) that did not hold up in replication studies [108, 109]. Our final approach, the negative control analyses, allowed us to maximize our sample size and representativeness (including participants without genotype information available) and ask whether postnatal exposures have similarly sized associations with outcomes measured prenatally. As with the other approaches, findings from this analysis alone would not be conclusive. Pre- and postnatal maternal stress are somewhat correlated, and adverse birth or early childhood outcomes may increase parental relationship stress over time. Both residual signal from prenatal exposure and reverse causation could bias our negative control estimates. Nonetheless, the minimal attenuation of associations in the negative control analyses (including postnatal life events, which are less susceptible to reverse causation), along with the consistency of these findings with the rest of our analyses, suggests that the selected exposures are likely to be valid negative controls.

Despite our best efforts to offset the limitations of our different approaches against one another, there are several over-arching limitations to consider. The participation rate in the MoBa cohort is 41%. Younger mothers, those with more than two previous births, single mothers, women with lower education levels, and smokers are under-represented relative to the target population. Additionally, the MoBa cohort is predominantly healthy, white, and of above-average socioeconomic status. MoBa participants with genotype data currently available for analysis are exclusively of European ancestry. Together, these considerations limit the generalizability of our results to diverse populations or settings. Another limitation is the reliance on self-reporting to measure maternal stress and maternal reporting of offspring difficulties. Self-reported data are prone to biases, such as recall bias, social desirability bias, and reporting bias. To mitigate these biases, where possible we used versions of standardized questionnaires validated and widely used in psychological research [67, 68, 73, 74]. Furthermore, it is also possible that self-report data captures women’s subjective experiences, emphasizing how they felt, which is key to understanding the impact stress had, which may offset the impact of any biases. A specific limitation of the adverse life event scale we used was that it referred to events during the past 12 months, thus capturing some stressors occurring before the prenatal period that was our focus.

While we use a broad definition of stress—including work-related stress, relationship dissatisfaction, and recent adverse life events, it does not capture all possible stressors—nor, due to the selected nature of the sample, do we necessarily capture the full range of variability among the domains we do study. Put otherwise, our focus on systematically quantifiable, normative stressors and the healthier-than-average nature of our cohort in Norway means we cannot evaluate the impact of either extreme stress—whether in these domains or elsewhere—or stress that is too unsystematic and idiosyncratic to be meaningfully measured by questionnaires [110]. We also did not distinguish between the timing of stress exposure during pregnancy, which could be important due to the varying vulnerability of developmental stages [100]. Finally, given that we largely rely on an absence of evidence to support our interpretations (though not exclusively, as evident in the negative control analyses), it is important to acknowledge that low statistical power can make it challenging to distinguish small effects from null effects. In the GxE and intergenerational MR analyses, in particular, limited precision in the estimates means that this is a consideration. Statistical power in these analyses is partly limited by the predictive capacity of our genetic instruments. For example, the neuroticism (*β* = 0.13, S.E. = 0.01, *R*^2^ = 0.02) and ADHD (*β* = 0.03, S.E. = 0.01, *R*^2^ < 0.01) PGS predicted corresponding trait measures in MoBa mothers relatively weakly, albeit robustly. Even though, in the GxE and MR analyses, the genetic instruments were used to tap factors not directly measured in MoBa mothers (sensitivity to stress and cortisol during pregnancy, respectively), it is clear that low power may significantly impact our findings.

## Conclusions

Using a triangulation approach of multiple methodologies, we found a consistent lack of evidence to support a causal link between prenatal maternal stress and early-life developmental outcomes in offspring. While our study does not nullify the possibility of causality, it underscores the likelihood that confounding factors will substantially inflate observational associations in this area. Our findings are restricted to the specific types of maternal stress exposures in MoBa. They should not be generalized to more extreme stressors, conditions, or different parts of the world [111]. Nonetheless, they should help reassure expectant mothers experiencing “everyday” stress.

## Supplementary Information

Additional file 1: sTables 1–6, sFigures 1–7, sMethods 1–2, and sEquation 1. sTable 1: Items included in scales assessing maternal work stress, relationship satisfaction, adverse life events, and child behavioral/emotional problems. sFigure 1: Path diagram illustrating the multilevel structural equation model used in sibling control analyses. sMethods 1: Details of inverse probability weighting for the sibling sub-sample in MoBa analyses. sFigure 2: Mean differences between siblings and singletons in the MoBa sample. sFigure 3: Estimation of sibling inclusion probabilities and weight adjustments in MoBa analysis. sFigure 4: Impact of inverse probability sampling weights on unweighted and weighted comparisons. sMethods 2: Assumptions and explanation of polygenic GxE analyses for inferring causal relationships. sEquation 1: Formal specification of the linear model used in polygenic GxE analyses. sFigure 5: Mendelian randomization (MR) results of maternal exposures on offspring outcomes without genotype adjustments. sFigure 6: Directed acyclic graph (DAG) of relationships tested in intergenerational MR analyses. sTable 2: Demographic characteristics of the study sample, including parental education, income, parity, and child sex. sTable 3: Mean differences in prenatal stress exposure by outcome data availability across waves. sFigure 7: Observational estimates of maternal stressors on offspring outcomes using full and weighted sibling samples. sTable 4: Parameter estimates from observational models evaluating maternal stress on offspring outcomes. sTable 5: Parameter estimates from polygenic GxE interaction models analyzing maternal stress and offspring outcomes. sTable 6: Parameter estimates from negative control models comparing maternal stressors with offspring outcomes.

## Data Availability

Data from the Norwegian Mother, Father, and Child Cohort Study and the Medical Birth Registry of Norway used in this study are managed by the national health register holders in Norway (Norwegian Institute of Public Health) and can be made available to researchers, provided approval from the Regional Committees for Medical and Health Research Ethics (REK), compliance with the EU General Data Protection Regulation (GDPR) and approval from the data owners. The consent given by the participants does not allow for data storage on an individual level in repositories or journals. Researchers who want access to data sets for replication should apply through helsedata.no. Access to data sets requires approval from The Regional Committee for Medical and Health Research Ethics in Norway. Code for data preparation and all analyses is available openly online at: https://github.com/psychgen/maternal-prenatal-stress.

## Abbreviations

ADHD: Attention deficit hyperactivity disorder
CBCL: Child Behavior Checklist
DAG: Directed acyclic graph
DoHAD: Developmental origins of health and disease
FDR: False discovery rate
GWAS: Genome-wide association studies
GxE: Genotype-environment interaction
MoBa: Norwegian Mother, Father, and Child Cohort Study
MR: Mendelian randomization
PGS: Polygenic score
PGS-PCA: Polygenic score-principal component analysis
PTSD: Post-traumatic stress disorder
SEM: Structural equation modeling
SES: Socioeconomic status
SNP: Single nucleotide polymorphism
