# Supplementary material for: Prenatal maternal stress: triangulating evidence for intrauterine exposure effects on birth and early childhood outcomes across multiple approaches
Source: BMC Med. 2025 Jan 21;23:18. doi: 10.1186/s12916-024-03834-w (PMC11753172; doi:10.1186/s12916-024-03834-w)
Supplement: Supplementary file 1 — Additional file 1: sTables 1–6, sFigures 1–7, sMethods 1–2, and sEquation 1. sTable 1: Items included in scales assessing maternal work stress, relationship satisfaction, adverse life events, and child behavioral/emotional problems. sFigure 1: Path diagram illustrating the multilevel structural equation model used in sibling control analyses. sMethods 1: Details of inverse probability weighting for the sibling sub-sample in MoBa analyses. sFigure 2: Mean differences between siblings and singletons in the MoBa sample. sFigure 3: Estimation of sibling inclusion probabilities and weight adjustments in MoBa analysis. sFigure 4: Impact of inverse probability sampling weights on unweighted and weighted comparisons. sMethods 2: Assumptions and explanation of polygenic GxE analyses for inferring causal relationships. sEquation 1: Formal specification of the linear model used in polygenic GxE analyses. sFigure 5: Mendelian randomization (MR) results of maternal exposures on offspring outcomes without genotype adjustments. sFigure 6: Directed acyclic graph (DAG) of relationships tested in intergenerational MR analyses. sTable 2: Demographic characteristics of the study sample, including parental education, income, parity, and child sex. sTable 3: Mean differences in prenatal stress exposure by outcome data availability across waves. sFigure 7: Observational estimates of maternal stressors on offspring outcomes using full and weighted sibling samples. sTable 4: Parameter estimates from observational models evaluating maternal stress on offspring outcomes. sTable 5: Parameter estimates from polygenic GxE interaction models analyzing maternal stress and offspring outcomes. sTable 6: Parameter estimates from negative control models comparing maternal stressors with offspring outcomes. [file 12916_2024_3834_MOESM1_ESM.docx]

Additional file 1 of Prenatal maternal stress: triangulating evidence for intrauterine exposure effects on birth and early childhood outcomes across multiple approaches

Table of Contents

[sTable 1. Items included in scales 2](#_Toc183767210)

[sFigure 1. Path diagram illustrating the multilevel structural equation model used in the sibling control analyses 4](#_Toc183767211)

[sMethods 1: Inverse probability weighting of sibling sub-sample 5](#_Toc183767212)

[sFigure 2. The mean difference between siblings and singletons (SDs) 5](#_Toc183767213)

[sFigure 3. Estimating sibling inclusion probability and weight adjustments in MoBa analysis 6](#_Toc183767214)

[sFigure 4. Impact of inverse probability of sampling weights on unweighted and weighted comparisons 7](#_Toc183767215)

[sMethods 2. Information about the use and assumptions of testing GxE to infer causality in observational relationships 8](#_Toc183767216)

[sEquation 1. Formal specification of the basic linear model used in the polygenic GxE analyses 9](#_Toc183767217)

[sFigure 5: MR results of maternal exposures on offspring outcomes without adjustment for offspring/partner genotype 10](#_Toc183767218)

[sFigure 6. Directed acyclic graph (DAG) of relationships tested and accounted for in the intergenerational MR analyses 11](#_Toc183767219)

[sTable 2. Demographic characteristics of the sample 12](#_Toc183767220)

[sTable 3: Mean differences in prenatal stress exposure by outcome data availability 13](#_Toc183767221)

[sFigure 7: Unadjusted observational estimates from both the full and (IPW weighted) sibling sample 14](#_Toc183767222)

[sTable 4. Parameter estimates from the observational models, full sample 15](#_Toc183767223)

[sTable 5. Parameter estimates from the polygenic GxE models 16](#_Toc183767224)

[sTable 6. Parameter estimates from the negative control models 20](#_Toc183767225)

sTable 1 outlines the items included in various scales used to assess maternal work stress, relationship satisfaction, adverse life events, and child behavioral and emotional problems at specific time points during and after pregnancy.

# sTable 1. Items included in scales

| **Measures/scales** | **Items** | **Response options** | |
| --- | --- | --- | --- |
| **Work stress** |  |  | |
| Indicate the appropriate answer for each of the following questions concerning your present work situation | Do you sometimes have so much to do that your work situation becomes taxing? ^a, b^ | 1 Yes, every day more than half of the working day  2-Yes, every day less than half of the working day”  3-Yes, periodically but not daily”  4-Seldom or never | |
|  | Are you subjected to a lot of uncomfortable background noise? ^a b^ |  |  |
|  |  |  | |
| How do the following statements describe your work situation? | My work is very stressful^a^ | 1-Agree  2-Agree mostly  3-Disagree mostly  4-Disagree | |
|  | My work demands a lot of me^a^ |  |  |
| **Relationship Satisfaction** | |  | |
| How well do these statements describe your relationship**?** | I have a close relationship with my spouse/partner ^a,b^ | 1-Agree completely  2-Agree  3-Agree somewhat  4-Disagree somewhat  5-Disagree  6-Disagree completely | |
|  | My partner and I have problems in our relationship ^a,b^ |  |  |
|  | I am very happy with our relationship ^a,b^ |  |  |
|  | My partner is generally understanding ^a,b^ |  |  |
|  | I often consider ending our relationship ^a,b^ |  |  |
|  | I am satisfied with my relationship with my partner ^a,b^ |  |  |
|  | We frequently disagree on important decisions ^a,b^ |  |  |
|  | I have been lucky in my choice of a partner ^a,b^ |  |  |
|  | We agree on how our child should be raised ^a,b^ |  |  |
|  | I believe my partner is satisfied with our relationship ^a,b^ |  |  |
| **Maternal adverse life events** | | | |
|  | Have you had problems at work or where you study?^b^ | 1 -No  2-Yes | *If yes:*  1-Not too bad  2-Painful/difficult 3-Very painful/difficult |
|  | Have you had financial problems? ^b^ |  |  |
|  | Have you been divorced, separated or ended the relationship with your partner? ^b^ |  |  |
|  | Have you had any problems or conflicts with your family, friends or neighbors? ^b^ |  |  |
|  | Have you been seriously ill or injured? ^b^ |  |  |
|  | Has anyone close to you been seriously ill or injured? ^b^ |  |  |
|  | Have you been involved in a serious traffic accident, house fire or robbery? ^b^ |  |  |
|  | Have you lost someone close to you? ^b^ |  |  |
|  | Other ^b^ |  |  |
| **Child Behaviour CheckList** | |  | |
| *Emotional problems* | Disturbed by any change in routine ^c, d, e^ | 1-Not true  2-Somewhat or sometimes true  3-Very true or often true | |
|  | Clings to adults or too dependent ^c, d, e^ |  |  |
|  | Gets too upset when separated from parents ^c,d,e^ |  |  |
|  | Too fearful or anxious ^c,d,e^ |  |  |
|  | Doesn’t eat well ^c,d,e^ |  |  |
|  | Sudden changes in moods or feelings ^d^ |  |  |
|  | Constipated, doesn’t move bowels ^d^ |  |  |
|  | Stomach aches or cramps (without medical cause) ^d^ |  |  |
|  | Vomiting, throwing up (without medical cause) ^d,e^ |  |  |
|  | Feelings are easily hurt^e^ |  |  |
|  | Nervous, high strung, or tense^e^ |  |  |
|  | Self-conscious or easily embarrassed_e_ |  |  |
|  | Unhappy, sad or depressed^e^ |  |  |
|  | Stomach aches or cramps (without medical cause) ^e^ |  |  |
| *Behavior problems* | Can’t concentrate, can’t pay attention for long ^c,d,e^ |  |  |
|  | Can’t sit still, restless or overactive ^c,d,e^ |  |  |
|  | Poorly coordinated or clumsy ^d,e^ |  |  |
|  | Quickly shifts from one activity to another ^c,d,e^ |  |  |
|  | Can’t stand waiting, wants everything now ^d,e^ |  |  |
|  | Defiant ^c,d,e^ |  |  |
|  | Demands must be met immediately ^d,e^ |  |  |
|  | Doesn’t seem to feel guilty after misbehaving ^c,d,e^ |  |  |
|  | Gets in many fights ^c,d,e^ |  |  |
|  | Hits others ^c,d,e^ |  |  |
|  | Punishment doesn’t change his/her behavior ^c,d,e^ |  |  |
| Notes: a= responded during pregnancy week 15, b =responded during pregnancy week 30, c= responded at child age 18 months, 30, d= responded at child age 3 years, e= responded at child age 5 years. | | | |

###

# sFigure 1. Path diagram illustrating the multilevel structural equation model used in the sibling control analyses


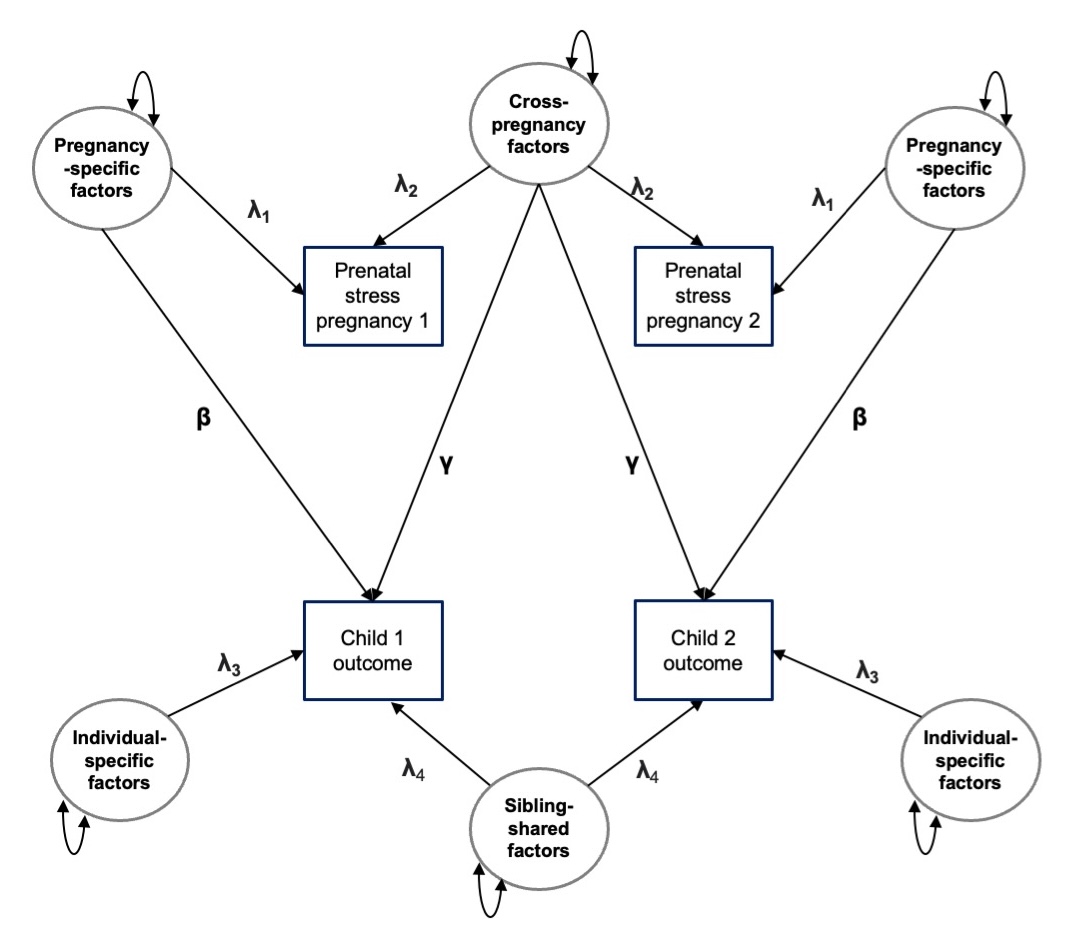


Notes: covariates are included in the models but not shown; models are extended to include simultaneous estimation of effects for emotional and behavioural child outcomes at all three included measurement occasions, but only the model for a single outcome is depicted here; the unadjusted results presented in Figure 2 in the manuscript are obtained by constraining the 𝝲 pathway to zero

# sMethods 1: Inverse probability weighting of sibling sub-sample

Due to participation bias, the sibling sub-sample of MoBa used for the sibling control analyses differs from the overall MoBa sample. sFigure 2 illustrates mean differences in the variables used in this study between the siblings and the remainder of the MoBa sample (here called “singletons,” although these also include families with multiple children where only 1 is a MoBa participant).

# sFigure 2. The mean difference between siblings and singletons (SDs)


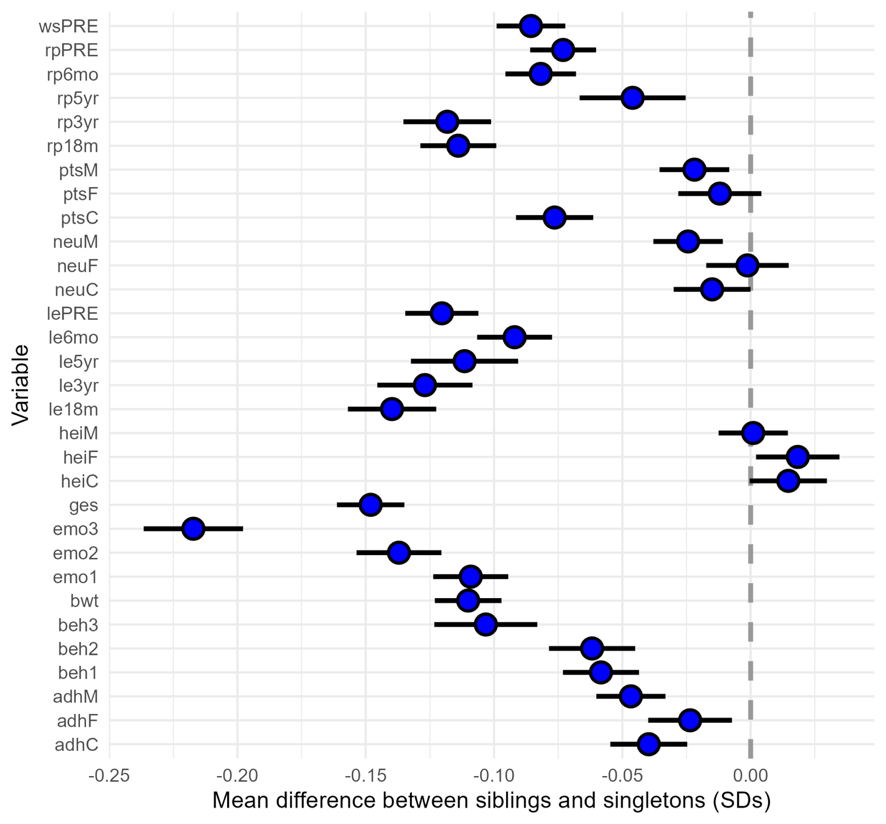


Notes: SD = Standard Deviations

By predicting sibling status using variables in our dataset, we can estimate MoBa individuals’ probability of being included in the sibling dataset and up-weight observations from those who are more similar to those not included, making estimates from analyses of this subset more similar to those that would have been obtained using the entire sample. The details of this approach are shown in the file 00.1_generate_sib_weights.R in the GitHub repository for this project (<https://github.com/psychgen/maternal-prenatal-stress>). The resulting smoothed inverse probability of sampling weights (IPSW) are linearly related to variables that index selection effects in the sub-sample, as shown in sFigure 3:

# sFigure 3. Estimating sibling inclusion probability and weight adjustments in MoBa analysis


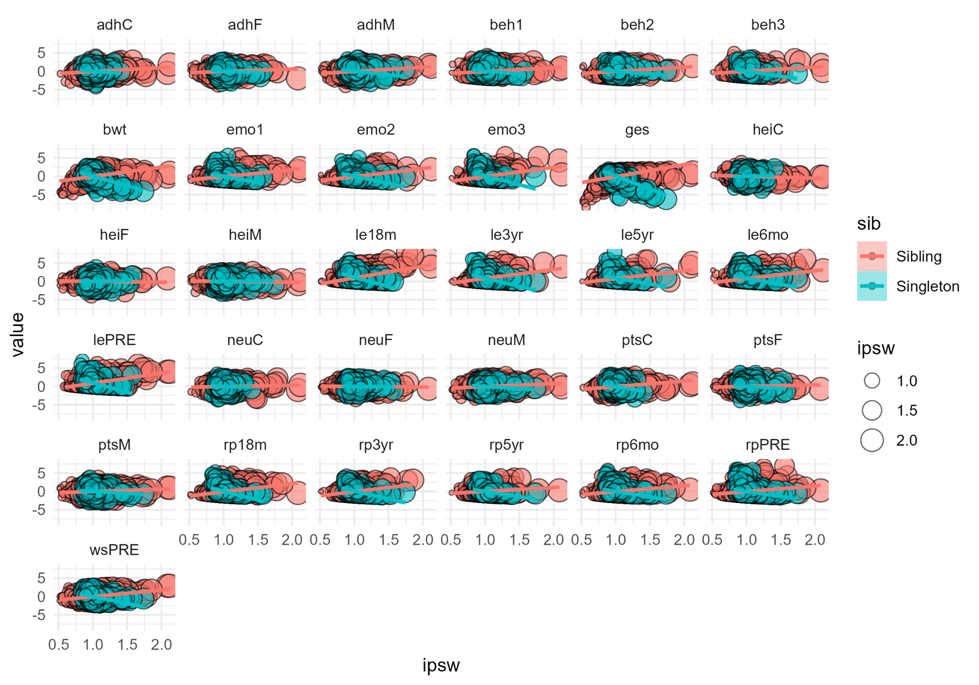


Notes: IPSW = inverse probability of sampling

Further, the impact of these weights on the comparisons shown in sFigure3 is shown in sFigure4.

# sFigure 4. Impact of inverse probability of sampling weights on unweighted and weighted comparisons


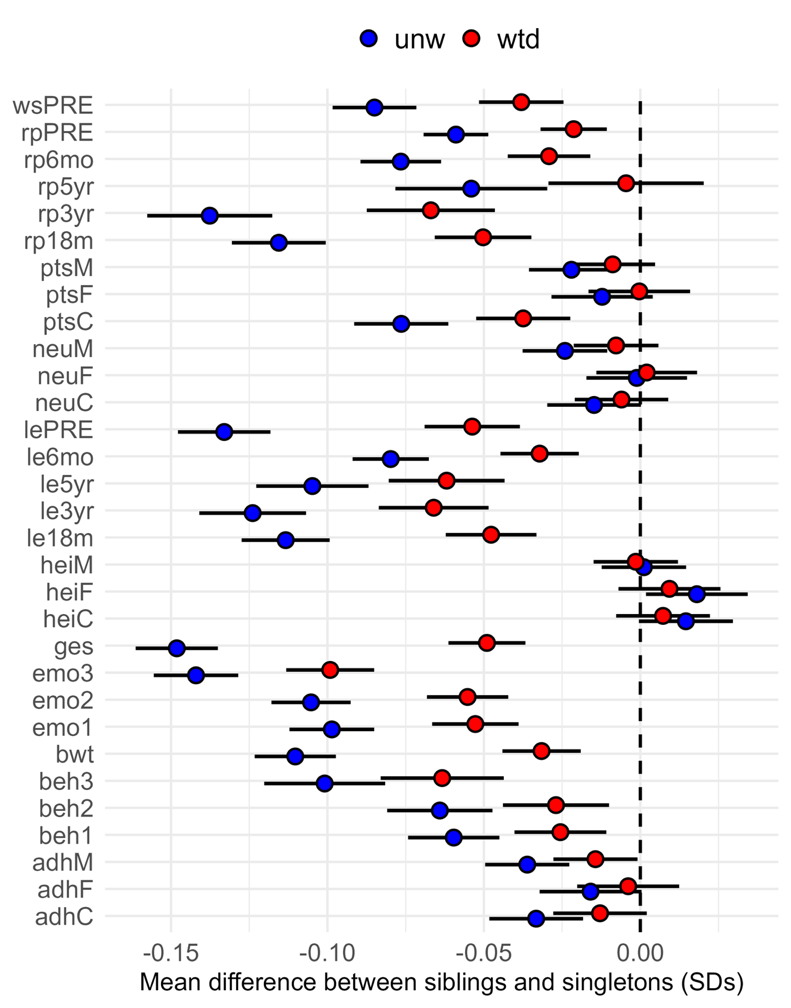


Notes: SD = standard Deviations, unw = unweighted, wtd = weighted

# sMethods 2. Information about the use and assumptions of testing GxE to infer causality in observational relationships

The inclusion of polygenic GxE analysis to infer causality in observed relationships between prenatal exposures and offspring outcomes warrants additional explanation. It relies on several assumptions of varying strength:

1. It is assumed that the polygenic scores included as moderators index (at least partially) mothers’ genetic sensitivity to environmental stress
2. It is assumed that the intrauterine environment will be more negatively influenced by prenatal stress experienced by mothers with greater genetic sensitivity to environmental stress.
3. It is assumed that the intrauterine environment will never be made more favorable by maternal exposure to prenatal stress, even among those with a lower genetic sensitivity to environmental stress (i.e., no cross-over interactions)
4. It is assumed that genetic sensitivity to environmental stress, as indexed by selected polygenic scores, will not moderate exposure-outcome associations arising due to non-causal mechanisms (e.g., shared genes, environments)

Assumption #1 is a particularly strong assumption because the method cannot provide indirect evidence of a causal effect - even if one exists - unless the selected moderators influence individual responsivity to stress. Assumption #4 is also quite strong since it assumes no non-causal mechanisms can produce a multiplicative interaction between an exposure and a moderator.

If each of these assumptions holds true, then evidence of a GxE interaction effect from these models would provide indirect evidence of a causal effect underpinning the observed exposure-outcome relationship. This is because the combination of #1, #2, and #3 above necessitates a main exposure-outcome effect in scenarios where a moderation effect is observed, and #4 necessitates that such an effect has a non-zero causal component.

These assumptions are difficult to test empirically because doing so would require a “positive control” exposure - i.e., data on an environmental stressor that definitively causally impacts the outcomes in question. Identifying such stressors from within measures of the environment typically available in cohort studies is an ongoing epidemiological challenge.

$${Child}_{outcome}=\beta_{0}+\beta_{1}m{Stress}_{1}+ \beta_{2}mPGS_{trait}+$$

$$\beta_{3}m{Stress}_{1}:mPGS_{trait}+ \beta_{4}cov_{1}+ \beta_{5}m{Stress}_{1}:cov_{1}+$$

$$\beta_{6}mPGS_{trait}:cov_{1}+\ldots+\varepsilon$$

# sEquation 1. Formal specification of the basic linear model used in the polygenic GxE analyses

Note: mStress= maternal prenatal stress; mPGS = maternal polygenic score; cov= covariate; “outcome” subscript denotes the specific outcome (birthweight, gestational age, behavioral problems, or emotional problems); “…” denotes a replication of the pattern of main effects and interactions for each included exposure and covariate; “trait” subscript denotes the PGS trait (either ADHD, neuroticism, PTSD, or height). Separate models were run for each *outcome* and PGS *trait*. For the child outcomes measured at multiple waves (child age 1.5, 3, or 5 years old), three versions of the model were run concurrently, and a constraint of both the main effects of exposures and their interactions with PGS across waves was tested using a likelihood ratio test

sFigure 5 presents the Mendelian Randomization (MR) results, focusing on maternal genotypic influences on offspring outcomes: emotional and behavioral responses at 1.5, 3, and 5 years, as well as gestational age and birthweight. The analysis is conducted without adjustments for offspring or partner genotype.

# sFigure 5: MR results of maternal exposures on offspring outcomes without adjustment for offspring/partner genotype


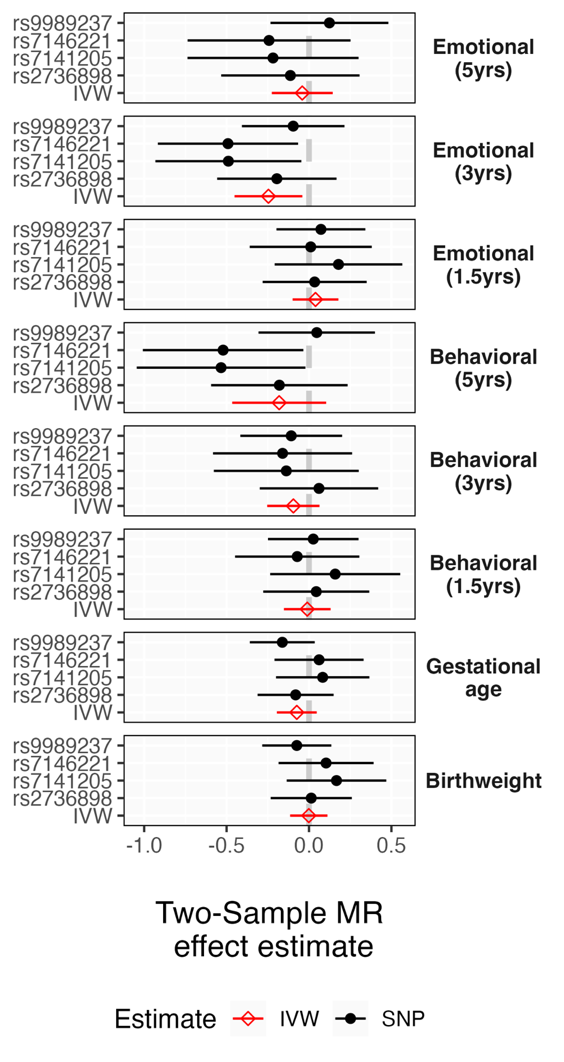


Notes: MR = Mendelian randomization, IVW= Inverse variance weighted, SNP = Single nucleotide polymorphism, rs= reference SNP , yrs= years

# sFigure 6. Directed acyclic graph (DAG) of relationships tested and accounted for in the intergenerational MR analyses

**
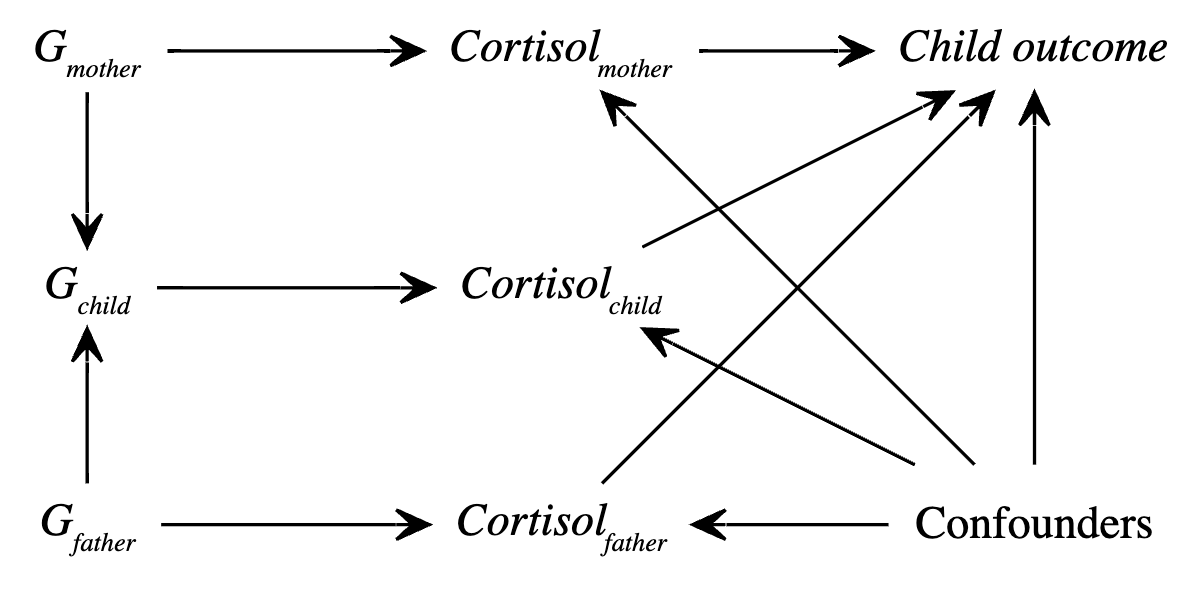
**

*Note – G represents a generic genetic instrument; in the case of our analyses, four separate cortisol-associated SNPs are used*

sTable 2 provides an overview of the demographic characteristics of the sample used in our study: maternal and paternal education, income levels, parity, and child sex, as well as the availability of maternal genotype data.

# sTable 2. Demographic characteristics of the sample

| **Characteristic** | **Level** | **N** | **%** |
| --- | --- | --- | --- |
| Maternal education | Elementary | 2754 | (2.4%) |
| Maternal education | University degree, <=4 years (Bachelors) | 39725 | (35.2%) |
| Maternal education | University degree, >4 years (Master’s, PhD) | 22695 | (20.1%) |
| Maternal education | Upper secondary, vocational, or other | 31970 | (28.3%) |
| Paternal education | Elementary | 4631 | (4.1%) |
| Paternal education | University degree, <=4 years (Bachelors) | 25302 | (22.4%) |
| Paternal education | University degree, >4 years (Master’s, PhD) | 21919 | (19.4%) |
| Paternal education | Upper secondary, vocational, or other | 41227 | (36.6%) |
| Maternal income | <150,000 NOK | 15529 | (13.8%) |
| Maternal income | >=500,000 NOK | 4467 | (4%) |
| Maternal income | 150-199,000 NOK | 11198 | (9.9%) |
| Maternal income | 200-299,000 NOK | 33590 | (29.8%) |
| Maternal income | 300-399,000 NOK | 24361 | (21.6%) |
| Maternal income | 400-499,000 NOK | 7052 | (6.3%) |
| Maternal income | None | 2522 | (2.2%) |
| Paternal income | <150,000 NOK | 5435 | (4.8%) |
| Paternal income | >=500,000 NOK | 15722 | (13.9%) |
| Paternal income | 150-199,000 NOK | 4164 | (3.7%) |
| Paternal income | 200-299,000 NOK | 21688 | (19.2%) |
| Paternal income | 300-399,000 NOK | 31061 | (27.5%) |
| Paternal income | 400-499,000 NOK | 16075 | (14.3%) |
| Paternal income | None | 984 | (0.9%) |
| Parity | 1st child | 49610 | (44%) |
| Parity | 2nd child | 40570 | (36%) |
| Parity | 3rd child | 17507 | (15.5%) |
| Parity | 4th child | 3875 | (3.4%) |
| Parity | 5th child (or later) | 1221 | (1.1%) |
| Child sex | Female | 54996 | (48.8%) |
| Child sex | Male | 57787 | (51.2%) |
| Maternal genotype data | Available | 93564 | (83%) |
| Maternal genotype data | Missing | 19220 | (17%) |

sTable 3 illustrates the evidence of selective attrition in the study sample by showing the mean differences in prenatal stress exposures between participants who provided outcome data and those who did not, at each wave of data collection. Small mean differences suggest minimal bias due to attrition in the study.

# sTable 3: Mean differences in prenatal stress exposure by outcome data availability

| **Outcome availability** | **Work stress (mean)** | **Work stress (sd)** | **Relationship stress (mean)** | **Relationship stress (sd)** | **Life events (mean)** | **Life events (sd)** |
| --- | --- | --- | --- | --- | --- | --- |
| Birth outcomes available | -0.002 | 0.999 | 0.000 | 0.999 | 0.946 | 1.084 |
| Birth outcomes missing | 0.075 | 1.264 | -0.052 | 0.961 | 0.655 | 0.775 |
| 1.5yr outcomes available | -0.053 | 0.978 | -0.039 | 0.952 | 0.92 | 1.062 |
| 1.5yr outcomes missing | 0.146 | 1.044 | 0.106 | 1.113 | 1.031 | 1.149 |
| 3yr outcomes available | -0.064 | 0.967 | -0.051 | 0.939 | 0.912 | 1.055 |
| 3yr outcomes missing | 0.081 | 1.035 | 0.069 | 1.073 | 0.995 | 1.123 |
| 5yr outcomes available | -0.076 | 0.961 | -0.062 | 0.944 | 0.896 | 1.046 |
| 5yr outcomes missing | 0.05 | 1.021 | 0.043 | 1.034 | 0.981 | 1.109 |

sFigure 7 shows unadjusted observational estimates of maternal stressors (life events, relationship problems and work stress) on offspring outcomes from both the full sample and the inverse probability weighted (IPW) sibling sample using a multilevel structural equation model (SEM).

# sFigure 7: Unadjusted observational estimates from both the full and (IPW weighted) sibling sample


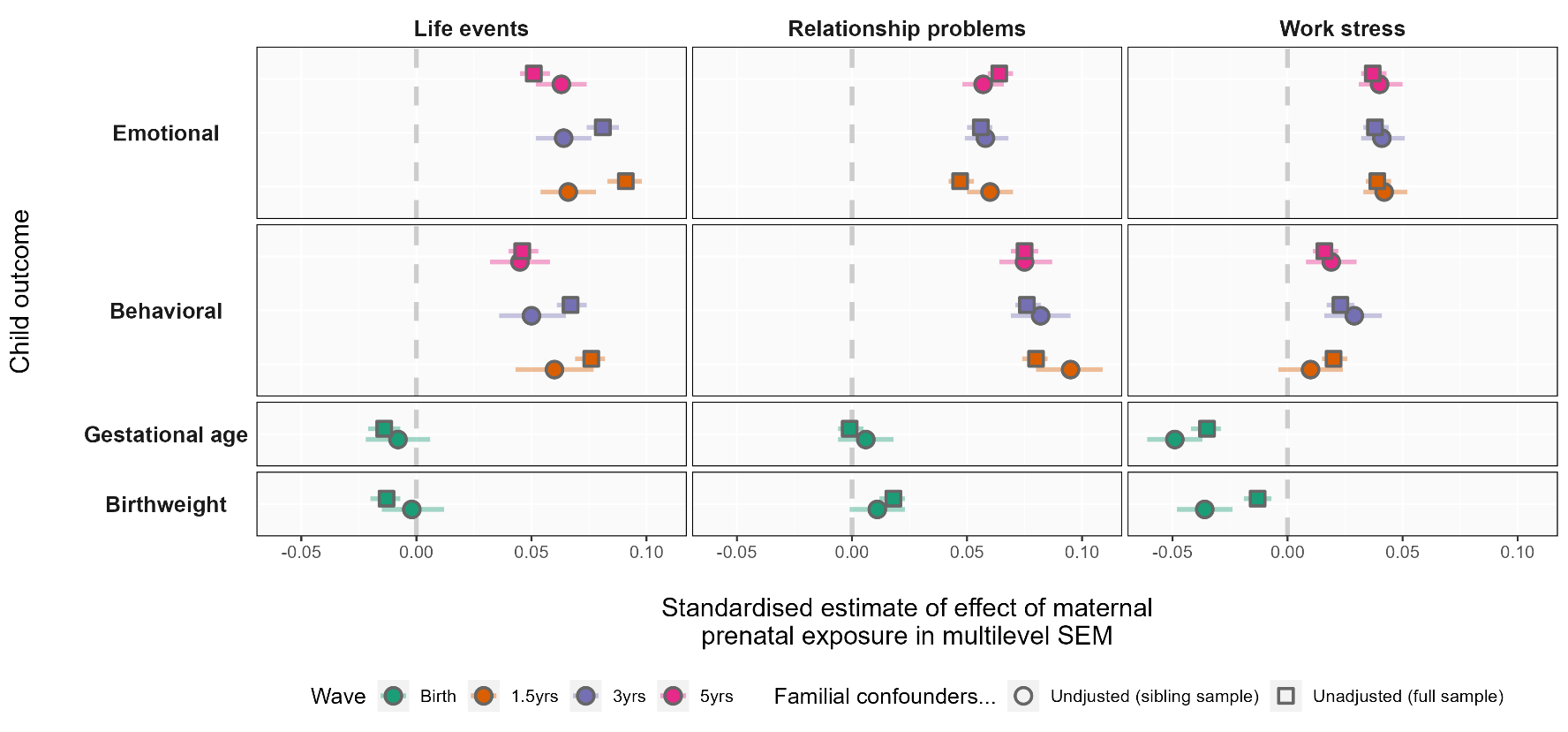


Notes: yrs= years

sTable 4 presents the parameter estimates from observational models assessing the impact of various types of maternal stress—work stress, relationship problems, and stressful life events—on different offspring outcomes at multiple time point

# sTable 4. Parameter estimates from the observational models, full sample

| **Maternal exposure** | **Offspring outcome** | **Wave** | **Estimate** | **Lower confidence interval** | **Upper confidence interval** |
| --- | --- | --- | --- | --- | --- |
| Work stress | Emotional | 1.5years | 0.039 | 0.034 | 0.045 |
| Relationship problems | Emotional | 1.5years | 0.047 | 0.042 | 0.053 |
| Life events | Emotional | 1.5years | 0.091 | 0.083 | 0.098 |
| Work stress | Emotional | 3years | 0.038 | 0.033 | 0.044 |
| Relationship problems | Emotional | 3years | 0.056 | 0.050 | 0.061 |
| Life events | Emotional | 3years | 0.081 | 0.074 | 0.088 |
| Work stress | Emotional | 5years | 0.037 | 0.032 | 0.043 |
| Relationship problems | Emotional | 5years | 0.064 | 0.059 | 0.070 |
| Life events | Emotional | 5years | 0.051 | 0.045 | 0.058 |
| Work stress | Behavioral | 1.5years | 0.020 | 0.015 | 0.026 |
| Relationship problems | Behavioral | 1.5years | 0.080 | 0.074 | 0.085 |
| Life events | Behavioral | 1.5years | 0.076 | 0.069 | 0.082 |
| Work stress | Behavioral | 3years | 0.023 | 0.017 | 0.029 |
| Relationship problems | Behavioral | 3years | 0.076 | 0.071 | 0.082 |
| Life events | Behavioral | 3years | 0.067 | 0.061 | 0.074 |
| Work stress | Behavioral | 5years | 0.016 | 0.011 | 0.022 |
| Relationship problems | Behavioral | 5years | 0.075 | 0.069 | 0.081 |
| Life events | Behavioral | 5years | 0.046 | 0.040 | 0.053 |
| Work stress | Gestational age | Birth | -0.035 | -0.042 | -0.029 |
| Relationship problems | Gestational age | Birth | -0.001 | -0.006 | 0.005 |
| Life events | Gestational age | Birth | -0.014 | -0.021 | -0.007 |
| Work stress | Birthweight | Birth | -0.013 | -0.019 | -0.007 |
| Relationship problems | Birthweight | Birth | 0.018 | 0.012 | 0.023 |
| Life events | Birthweight | Birth | -0.013 | -0.020 | -0.007 |

sTable 5 presents the parameter estimates from polygenic Gene-by-Environment (GxE) interaction models. This table details the effects of maternal stress exposures—such as work stress, relationship problems, and life events—on offspring outcomes, including birthweight, gestational age, and emotional and behavioral problems at various developmental stages (birth, 18 months, three years, and five years).

# sTable 5. Parameter estimates from the polygenic GxE models

| **Maternal exposures** | **Offspring outcomes** | **Wave** | **PGS moderator** | **Std. interaction effect** | **Lower confidence interval** | **Upper confidence interval** | **FDR-corrected p-value** |
| --- | --- | --- | --- | --- | --- | --- | --- |
| Work stress | Birthweight | Birth | Neurot. | 0.003 | -0.005 | 0.011 | 0.874 |
| Relationship problems | Birthweight | Birth | Neurot. | -0.007 | -0.015 | 0.001 | 0.598 |
| Life events | Birthweight | Birth | Neurot. | 0.002 | -0.006 | 0.009 | 0.879 |
| Work stress | Gestational age | Birth | Neurot. | 0.003 | -0.005 | 0.012 | 0.874 |
| Relationship problems | Gestational age | Birth | Neurot. | -0.006 | -0.014 | 0.002 | 0.610 |
| Life events | Gestational age | Birth | Neurot. | 0.003 | -0.005 | 0.012 | 0.874 |
| Work stress | Emotional | 5years | Neurot. | 0.001 | -0.008 | 0.010 | 0.967 |
| Relationship problems | Emotional | 5years | Neurot. | -0.003 | -0.012 | 0.007 | 0.875 |
| Life events | Emotional | 5years | Neurot. | -0.004 | -0.013 | 0.004 | 0.838 |
| Work stress | Emotional | 3years | Neurot. | 0.006 | -0.004 | 0.016 | 0.835 |
| Relationship problems | Emotional | 3years | Neurot. | 0.000 | -0.010 | 0.011 | 0.969 |
| Life events | Emotional | 3years | Neurot. | -0.004 | -0.013 | 0.005 | 0.874 |
| Work stress | Emotional | 1.5years | Neurot. | -0.007 | -0.018 | 0.005 | 0.838 |
| Relationship problems | Emotional | 1.5years | Neurot. | 0.001 | -0.011 | 0.012 | 0.969 |
| Life events | Emotional | 1.5years | Neurot. | 0.010 | -0.001 | 0.021 | 0.598 |
| Work stress | Behavioral | 5years | Neurot. | -0.006 | -0.013 | 0.001 | 0.598 |
| Relationship problems | Behavioral | 5years | Neurot. | 0.000 | -0.008 | 0.008 | 0.969 |
| Life events | Behavioral | 5years | Neurot. | -0.002 | -0.009 | 0.004 | 0.874 |
| Work stress | Behavioral | 3years | Neurot. | -0.006 | -0.013 | 0.001 | 0.598 |
| Relationship problems | Behavioral | 3years | Neurot. | 0.000 | -0.008 | 0.008 | 0.969 |
| Life events | Behavioral | 3years | Neurot. | -0.002 | -0.009 | 0.004 | 0.874 |
| Work stress | Behavioral | 1.5years | Neurot. | -0.006 | -0.013 | 0.001 | 0.598 |
| Relationship problems | Behavioral | 1.5years | Neurot. | 0.000 | -0.008 | 0.008 | 0.969 |
| Life events | Behavioral | 1.5years | Neurot. | -0.002 | -0.009 | 0.004 | 0.874 |
| Work stress | Birthweight | Birth | PTSD | -0.008 | -0.016 | 0.000 | 0.598 |
| Relationship problems | Birthweight | Birth | PTSD | 0.003 | -0.004 | 0.011 | 0.874 |
| Life events | Birthweight | Birth | PTSD | -0.004 | -0.011 | 0.004 | 0.870 |
| Work stress | Gestational age | Birth | PTSD | -0.005 | -0.013 | 0.004 | 0.838 |
| Relationship problems | Gestational age | Birth | PTSD | 0.006 | -0.002 | 0.014 | 0.610 |
| Life events | Gestational age | Birth | PTSD | -0.007 | -0.015 | 0.002 | 0.610 |
| Work stress | Emotional | 5years | PTSD | 0.003 | -0.006 | 0.011 | 0.875 |
| Relationship problems | Emotional | 5years | PTSD | 0.000 | -0.010 | 0.009 | 0.969 |
| Life events | Emotional | 5years | PTSD | -0.002 | -0.010 | 0.006 | 0.879 |
| Work stress | Emotional | 3years | PTSD | 0.004 | -0.007 | 0.014 | 0.874 |
| Relationship problems | Emotional | 3years | PTSD | -0.008 | -0.018 | 0.002 | 0.610 |
| Life events | Emotional | 3years | PTSD | -0.003 | -0.012 | 0.006 | 0.874 |
| Work stress | Emotional | 1.5years | PTSD | -0.007 | -0.019 | 0.005 | 0.838 |
| Relationship problems | Emotional | 1.5years | PTSD | -0.013 | -0.025 | 0.000 | 0.598 |
| Life events | Emotional | 1.5years | PTSD | -0.003 | -0.014 | 0.008 | 0.875 |
| Work stress | Behavioral | 5years | PTSD | 0.000 | -0.008 | 0.009 | 0.969 |
| Relationship problems | Behavioral | 5years | PTSD | -0.003 | -0.012 | 0.006 | 0.874 |
| Life events | Behavioral | 5years | PTSD | 0.002 | -0.005 | 0.010 | 0.875 |
| Work stress | Behavioral | 3years | PTSD | 0.001 | -0.008 | 0.011 | 0.915 |
| Relationship problems | Behavioral | 3years | PTSD | 0.009 | -0.002 | 0.019 | 0.598 |
| Life events | Behavioral | 3years | PTSD | -0.002 | -0.011 | 0.007 | 0.879 |
| Work stress | Behavioral | 1.5years | PTSD | 0.008 | -0.003 | 0.020 | 0.707 |
| Relationship problems | Behavioral | 1.5years | PTSD | 0.016 | 0.003 | 0.029 | 0.598 |
| Life events | Behavioral | 1.5years | PTSD | 0.002 | -0.009 | 0.012 | 0.894 |
| Work stress | Birthweight | Birth | ADHD | 0.001 | -0.007 | 0.009 | 0.967 |
| Relationship problems | Birthweight | Birth | ADHD | 0.001 | -0.007 | 0.008 | 0.967 |
| Life events | Birthweight | Birth | ADHD | -0.003 | -0.010 | 0.005 | 0.874 |
| Work stress | Gestational age | Birth | ADHD | 0.004 | -0.004 | 0.013 | 0.838 |
| Relationship problems | Gestational age | Birth | ADHD | 0.004 | -0.003 | 0.012 | 0.838 |
| Life events | Gestational age | Birth | ADHD | -0.003 | -0.011 | 0.005 | 0.874 |
| Work stress | Emotional | 5years | ADHD | -0.002 | -0.011 | 0.007 | 0.879 |
| Relationship problems | Emotional | 5years | ADHD | -0.005 | -0.014 | 0.005 | 0.846 |
| Life events | Emotional | 5years | ADHD | 0.002 | -0.006 | 0.010 | 0.879 |
| Work stress | Emotional | 3years | ADHD | 0.001 | -0.009 | 0.011 | 0.967 |
| Relationship problems | Emotional | 3years | ADHD | 0.003 | -0.007 | 0.013 | 0.875 |
| Life events | Emotional | 3years | ADHD | -0.003 | -0.012 | 0.006 | 0.874 |
| Work stress | Emotional | 1.5years | ADHD | -0.002 | -0.014 | 0.009 | 0.879 |
| Relationship problems | Emotional | 1.5years | ADHD | 0.003 | -0.009 | 0.015 | 0.879 |
| Life events | Emotional | 1.5years | ADHD | 0.001 | -0.011 | 0.012 | 0.969 |
| Work stress | Behavioral | 5years | ADHD | 0.005 | -0.003 | 0.014 | 0.835 |
| Relationship problems | Behavioral | 5years | ADHD | -0.013 | -0.023 | -0.004 | 0.437 |
| Life events | Behavioral | 5years | ADHD | -0.004 | -0.012 | 0.004 | 0.838 |
| Work stress | Behavioral | 3years | ADHD | -0.002 | -0.012 | 0.008 | 0.879 |
| Relationship problems | Behavioral | 3years | ADHD | 0.003 | -0.007 | 0.014 | 0.874 |
| Life events | Behavioral | 3years | ADHD | 0.002 | -0.007 | 0.011 | 0.879 |
| Work stress | Behavioral | 1.5years | ADHD | -0.001 | -0.012 | 0.010 | 0.967 |
| Relationship problems | Behavioral | 1.5years | ADHD | 0.004 | -0.008 | 0.016 | 0.874 |
| Life events | Behavioral | 1.5years | ADHD | -0.001 | -0.012 | 0.009 | 0.962 |
| Work stress | Birthweight | Birth | Height | -0.005 | -0.013 | 0.004 | 0.838 |
| Relationship problems | Birthweight | Birth | Height | -0.005 | -0.013 | 0.003 | 0.835 |
| Life events | Birthweight | Birth | Height | 0.001 | -0.006 | 0.009 | 0.879 |
| Work stress | Gestational age | Birth | Height | -0.005 | -0.013 | 0.003 | 0.838 |
| Relationship problems | Gestational age | Birth | Height | -0.005 | -0.013 | 0.003 | 0.835 |
| Life events | Gestational age | Birth | Height | 0.004 | -0.005 | 0.012 | 0.874 |
| Work stress | Emotional | 5years | Height | -0.007 | -0.016 | 0.002 | 0.610 |
| Relationship problems | Emotional | 5years | Height | 0.004 | -0.005 | 0.013 | 0.874 |
| Life events | Emotional | 5years | Height | 0.003 | -0.005 | 0.012 | 0.874 |
| Work stress | Emotional | 3years | Height | -0.005 | -0.014 | 0.005 | 0.874 |
| Relationship problems | Emotional | 3years | Height | 0.003 | -0.007 | 0.013 | 0.874 |
| Life events | Emotional | 3years | Height | -0.002 | -0.012 | 0.007 | 0.875 |
| Work stress | Emotional | 1.5years | Height | -0.004 | -0.017 | 0.008 | 0.874 |
| Relationship problems | Emotional | 1.5years | Height | 0.001 | -0.011 | 0.013 | 0.967 |
| Life events | Emotional | 1.5years | Height | -0.001 | -0.012 | 0.011 | 0.969 |
| Work stress | Behavioral | 5years | Height | -0.007 | -0.014 | 0.000 | 0.598 |
| Relationship problems | Behavioral | 5years | Height | -0.002 | -0.010 | 0.006 | 0.875 |
| Life events | Behavioral | 5years | Height | 0.006 | 0.000 | 0.013 | 0.598 |
| Work stress | Behavioral | 3years | Height | -0.007 | -0.014 | 0.000 | 0.598 |
| Relationship problems | Behavioral | 3years | Height | -0.002 | -0.010 | 0.006 | 0.875 |
| Life events | Behavioral | 3years | Height | 0.006 | 0.000 | 0.013 | 0.598 |
| Work stress | Behavioral | 1.5years | Height | -0.007 | -0.014 | 0.000 | 0.598 |
| Relationship problems | Behavioral | 1.5years | Height | -0.002 | -0.010 | 0.006 | 0.875 |
| Life events | Behavioral | 1.5years | Height | 0.006 | 0.000 | 0.013 | 0.598 |

Notes: FDR = false discovery rate, Neurot= Neuroticism, ADHD = Attention deficit hyperactivity disorder, PTSD = Post-traumatic stress disorder.

sTable 6 provides parameter estimates from negative control models alongside traditional exposure-outcome models, examining the effects of maternal stressors such as relationship problems and life events on various child outcomes at different stages.

# sTable 6. Parameter estimates from the negative control models

| **Maternal exposure** | **Exposure wave** | **Offspring outcome** | **Outcome wave** | **Model type** | **Estimate** | **Lower confidence interval** | **Upper confidence interval** |
| --- | --- | --- | --- | --- | --- | --- | --- |
| Relationship problems | Prenatal | Emotional | 5years | Exposure-outcome | 0.122 | 0.113 | 0.130 |
| Relationship problems | Prenatal | Emotional | 3years | Exposure-outcome | 0.130 | 0.120 | 0.139 |
| Relationship problems | 3years | Emotional | 5years | Negative control | 0.111 | 0.101 | 0.120 |
| Relationship problems | 5years | Emotional | 5years | Negative control | 0.092 | 0.080 | 0.103 |
| Relationship problems | 5years | Emotional | 3years | Negative control | 0.126 | 0.114 | 0.137 |
| Relationship problems | Prenatal | Behavioral | 5years | Exposure-outcome | 0.103 | 0.095 | 0.112 |
| Relationship problems | Prenatal | Behavioral | 3years | Exposure-outcome | 0.104 | 0.094 | 0.114 |
| Relationship problems | 3years | Behavioral | 5years | Negative control | 0.098 | 0.088 | 0.108 |
| Relationship problems | 5years | Behavioral | 5years | Negative control | 0.083 | 0.071 | 0.095 |
| Relationship problems | 5years | Behavioral | 3years | Negative control | 0.104 | 0.091 | 0.116 |
| Relationship problems | Prenatal | Gestational age | Birth | Exposure-outcome | 0.001 | -0.006 | 0.008 |
| Relationship problems | 1.5years | Gestational age | Birth | Negative control | -0.003 | -0.011 | 0.006 |
| Relationship problems | 3years | Gestational age | Birth | Negative control | -0.011 | -0.021 | -0.002 |
| Relationship problems | 5years | Gestational age | Birth | Negative control | -0.010 | -0.021 | 0.002 |
| Relationship problems | Prenatal | Birthweight | Birth | Exposure-outcome | 0.001 | -0.006 | 0.008 |
| Relationship problems | 1.5years | Birthweight | Birth | Negative control | -0.006 | -0.015 | 0.002 |
| Relationship problems | 3years | Birthweight | Birth | Negative control | -0.015 | -0.024 | -0.005 |
| Relationship problems | 5years | Birthweight | Birth | Negative control | -0.016 | -0.027 | -0.005 |
| Life events | Prenatal | Emotional | 5years | Exposure-outcome | 0.051 | 0.044 | 0.058 |
| Life events | Prenatal | Emotional | 3years | Exposure-outcome | 0.072 | 0.063 | 0.080 |
| Life events | 3years | Emotional | 5years | Negative control | 0.052 | 0.043 | 0.060 |
| Life events | 5years | Emotional | 5years | Negative control | 0.060 | 0.050 | 0.070 |
| Life events | 5years | Emotional | 3years | Negative control | 0.077 | 0.066 | 0.088 |
| Life events | Prenatal | Behavioral | 5years | Exposure-outcome | 0.061 | 0.054 | 0.069 |
| Life events | Prenatal | Behavioral | 3years | Exposure-outcome | 0.092 | 0.083 | 0.100 |
| Life events | 3years | Behavioral | 5years | Negative control | 0.069 | 0.061 | 0.078 |
| Life events | 5years | Behavioral | 5years | Negative control | 0.072 | 0.061 | 0.083 |
| Life events | 5years | Behavioral | 3years | Negative control | 0.106 | 0.094 | 0.117 |
| Life events | Prenatal | Gestational age | Birth | Exposure-outcome | -0.016 | -0.024 | -0.009 |
| Life events | 1.5years | Gestational age | Birth | Negative control | -0.026 | -0.033 | -0.018 |
| Life events | 3years | Gestational age | Birth | Negative control | -0.021 | -0.030 | -0.013 |
| Life events | 5years | Gestational age | Birth | Negative control | -0.026 | -0.037 | -0.015 |
| Life events | Prenatal | Birthweight | Birth | Exposure-outcome | -0.010 | -0.017 | -0.004 |
| Life events | 1.5years | Birthweight | Birth | Negative control | -0.012 | -0.019 | -0.005 |
| Life events | 3years | Birthweight | Birth | Negative control | -0.004 | -0.013 | 0.004 |
| Life events | 5years | Birthweight | Birth | Negative control | -0.012 | -0.022 | -0.001 |
